# Telomeric retrotransposons show propensity to form G-quadruplexes in various eukaryotic species

**DOI:** 10.1186/s13100-023-00291-9

**Published:** 2023-04-10

**Authors:** Pavel Jedlička, Viktor Tokan, Iva Kejnovská, Roman Hobza, Eduard Kejnovský

**Affiliations:** 1 Department of Plant Developmental Genetics, Institute of Biophysics of the Czech Academy of Sciences, Kralovopolska 135, 61200 Brno, Czech Republic; 2 Department of Biophysics of Nucleic Acids, Institute of Biophysics of the Czech Academy of Sciences, Kralovopolska 135, 61200 Brno, Czech Republic

**Keywords:** Telomere, G-quadruplex, Retrotransposon, *Drosophila*, Het-A, TART, TAHRE

## Abstract

**Background:**

Canonical telomeres (telomerase-synthetised) are readily forming G-quadruplexes (G4) on the G-rich strand. However, there are examples of non-canonical telomeres among eukaryotes where telomeric tandem repeats are invaded by specific retrotransposons. *Drosophila melanogaster* represents an extreme example with telomeres composed solely by three retrotransposons—Het-A, TAHRE and TART (HTT). Even though non-canonical telomeres often show strand biased G-distribution, the evidence for the G4-forming potential is limited.

**Results:**

Using circular dichroism spectroscopy and UV absorption melting assay we have verified in vitro G4-formation in the HTT elements of *D. melanogaster*. Namely 3 in Het-A, 8 in TART and 2 in TAHRE. All the G4s are asymmetrically distributed as in canonical telomeres. Bioinformatic analysis showed that asymmetric distribution of potential quadruplex sequences (PQS) is common in telomeric retrotransposons in other Drosophila species. Most of the PQS are located in the *gag* gene where PQS density correlates with higher DNA sequence conservation and codon selection favoring G4-forming potential. The importance of G4s in non-canonical telomeres is further supported by analysis of telomere-associated retrotransposons from various eukaryotic species including green algae, *Diplomonadida*, fungi, insects and vertebrates. Virtually all analyzed telomere-associated retrotransposons contained PQS, frequently with asymmetric strand distribution. Comparison with non-telomeric elements showed independent selection of PQS-rich elements from four distinct LINE clades.

**Conclusion:**

Our findings of strand-biased G4-forming motifs in telomere-associated retrotransposons from various eukaryotic species support the G4-formation as one of the prerequisites for the recruitment of specific retrotransposons to chromosome ends and call for further experimental studies.

**Supplementary Information:**

The online version contains supplementary material available at 10.1186/s13100-023-00291-9.

## Background

Chromosome ends need to be distinguished from double strand breaks. In eukaryotic organisms they are protected by telomeres, a complex of proteins and sequences that prevent chromosome fusion and the loss of coding DNA through incomplete replication of linear DNA. Telomeric sequences of most eukaryotic organisms consist of telomerase-synthesized head-to-tail short tandem repeats. In some species insertions of specific transposable elements into telomeric tandem repeats have been described, namely the SART and TRAS elements in *Bombyx mori* and *Tribolium castaneum* [1–3], GilM and GilT elements in *Giardia intestinalis* [4], Zepp1 element in *Chlorella vulgaris* [5], MoTeR1 element in *Pyricularia oryzae* [6] and Tx1-1_ACar in the anole lizard [7]. In these species the telomeric sequences are formed by both telomerase and transposable elements, however in some cases the telomerase activity is weak or absent hence the transposons possibly take over chromosome elongation function.

In *Drosophila* fruit fly species the telomerase is missing and telomeric sequences are formed solely by arrays of non-LTR retrotransposons and hence represent a unique chromosome end maintenance system [8, 9]. Telomeres of *Drosophila melanogaster* consist of three retrotransposons; Het-A, TART and TAHRE, that form a HTT array [8]. The individual retrotransposons are always oriented by the poly-A tails toward the centromere. These elements are fast evolving and specific to the *melanogaster* subgroup while telomeres of other *Drosophila* species are formed by other ancestrally related retrotransposons or, in the case of *D. biarmipes*, rely on recombination-based mechanisms similar to those of *Chironomus* [10, 11].

Telomere sequences of almost all studied eukaryotic organisms have the propensity to form four-stranded structures called G-quadruplexes (G4) [12]. This is due to the fact that repeat units often contain 3 consecutive guanines, a prerequisite for G4 formation. Since the G4-forming propensity of telomeric sequences is so widespread, these sequences became one of the golden standards to study both G4 in vitro formation and in vivo biological functions. There is some evidence for both positive (capping, telomerase stimulation, recombination based elongation) and negative (replication stalling, genome instability) aspects of G4 in telomere biology (reviewed in [13]). Nevertheless, it is not an easy task to monitor formation of particular G4 in vivo to infer its function. Hence the contribution of G4 to telomere maintenance is still not clear [13]. Similarly to canonical telomeres, the elements from the HTT array show composition bias making one strand G-rich [14]. It should be noted that this bias is subtle compared to canonical telomeres and as far as we are aware, its significance is not known (even for canonical telomeres). However, it was shown that the 3´ UTR of the Het-A element contains a sequence capable of G4 formation on the G-rich strand [15]. Even though the function of the G4 was not studied for the element nor the telomere, the G4-formation may possibly represent the explanation for the strand composition bias, shared by both canonical and non-canonical telomeres. Since the only study regarding G4 formation in telomere-associated retrotransposons is more than 20 years old [15], we wondered if up-to-date prediction methods could expand the evidence of G4-potential in non-canonical telomeres. Such observation could support the importance of G4 structures at non-canonical telomeres and provide clues to point out the common functions of G4 shared with canonical telomeres. Moreover it could also support the idea that G4s not only play a role in TE life-cycle but TEs can also serve as a vehicle for G4 spreading in the genomes [16]. However, a detailed analysis of G4-forming potential by telomeric retrotransposons is needed.

Here, we present a comprehensive study of the G4-forming potential of telomeric retrotransposons in various species, with special emphasis on *Drosophila*. We analyzed (i) the abundance and distribution of potential quadruplex-forming sequences (PQS) in telomeric HTT elements of *D. melanogaster*, (ii) the ability of identified PQS to adopt quadruplex conformation in vitro by circular dichroism analysis, (iii) the PQS strand distribution and abundance of PQS in telomeric retrotransposons in other *Drosophila* species, (vi) the DNA/amino acid identity bias with respect to PQS abundance and (v) PQS abundance and distribution in telomeric retrotransposons in other species also possessing telomerase-synthesized tandem arrays.

## Results

### HTT elements of *D. melanogaster* contain G4 motifs with strand-asymmetric distribution

In order to search for sequences with G4-forming potential (potential quadruplex-forming sequences, PQS) we used the pqsfinder software [17] on the reference Het-A, TART and TAHRE elements of *D. melanogaster* (Additional file 1: Fig. S1A-E). Altogether we identified 17 PQS with scores ranging between 47–73. The higher the score the better the chance of G4-formation in vitro is, for a score of 47 there is a 75% match in G4-formation with G4-seq data [18]. In particular we found 4 PQS in each Het-A and TAHRE and up to 9 PQS in TART. The number of PQS in TART elements depended on the subfamily. There were 8 PQS in TART-B, 7 PQS in TART-C and 6 PQS in TART-A. The TART-B contained PQS on all the positions as TART-A and TART-C with various sequence similarity (Additional file 1: Fig. S1C). The only exception was PQS in the *pol* gene of TART-A that was not present in TART-B in the respective position. For the following in vitro measurements we used the PQS from TART-B with the additional one from TART-A.

We also analyzed the consensus sequences of 5 Het-A subfamilies that reflect the intraspecific variability (Additional file 1: Fig S1F; [19]). We found that the PQS located in 3´UTR was the least conserved, missing in 3 subfamilies. The first PQS in the *gag* gene was present in all subfamilies and hence most conservative. Each of the remaining 2 PQS were missing in one subfamily. All PQSs showed strand-asymmetric distribution inside the HTT elements. These G-rich motifs were located on the non-coding strand (in relation to the *gag* and *pol* genes) and hence were not transcribed as a part of the sense transcript. Since HTT elements were arranged head-to-tail in the telomeric array with the poly-A tails toward centromere, all the PQS were present only on one strand just as in canonical telomeres.

**Fig. 1:**
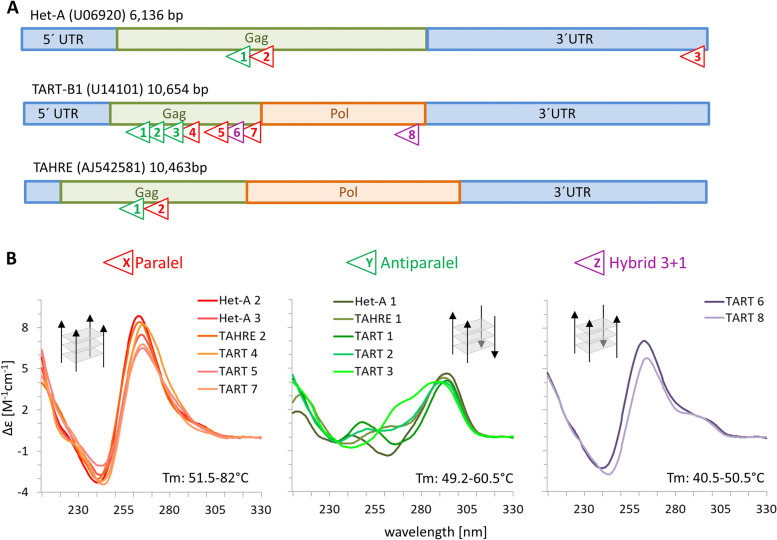
G4 distribution and experimental validation in the reference telomeric elements of *D. melanogaster*. **A** Schematic localization of G4 forming motifs inside Het-A, TART and TAHRE elements. Each triangle represents one in vitro verified G4. Orientation of the triangle indicates the PQS orientation (all G-rich sequences are on the template strand) and color indicates the topology (red for parallel, green for antiparallel and purple for 3 + 1 hybrid G4). Note that PQS8 in TART originates from TART-A1 (AY561850) at the respective position. The elements are not to scale. **B** CD spectra of all G4-forming sequences in 150 mM K^+^, melting temperature range as well as a visual representation of strand orientation is indicated for each topology

Using circular dichroism (CD) spectroscopy and UV absorption DNA melting assay we tested the G4 forming capability and G4 stability of predicted sequences. Circular dichroism is an optical spectroscopical method sensitively reflecting DNA conformation [20]. The method is based on measuring the difference of rotation of left- and right- circularly polarized light by DNA in solution. It is empirically known that the topology of parallel-stranded G4 (all strands run in the same direction, depicted in Fig. 1B) corresponds to the high positive band at 260 nm in CD spectrum while antiparallel-stranded G4 (2 strands run 5´-3´ and 2 in opposite direction) corresponds to the positive band at 295 nm and negative band at 260 nm [20]. A hybrid 3 + 1 (3 strands are parallel, one is antiparallel) topology is then reflected by a combination of the bands on the CD spectrum.

The UV absorption melting assay measures the decrease in absorbance at 297 nm, where G4 absorbs differently with changing temperature allowing to determine the G4 stability reflected by melting temperature [21]. Out of the 17 identified PQS, 13 formed G4 in vitro including one previously described in 3´ UTR of Het-A [15] (Fig. 1, Additional file 1: Fig. S1A,B,E,G). The other reported G4-forming sequence matched 3´ UTR that is upstream of Het-A mobile element in the accession number U06920 (Additional file 1: Fig. S1E). Three quadruplex-forming motifs were found in Het-A, eight in TART and two in TAHRE element. Except for the 3´ UTR located G4 in Het-A all other G4s were formed in the coding region and in particular within the *gag* gene. In terms of G4 topology, six oligonucleotides formed parallel-stranded G4s, five oligonucleotides formed antiparallel-stranded G4 and two oligonucleotides adopted a 3 + 1 hybrid conformation (Fig. 1B). The topologies are ordered according to a decreasing average thermal stability with melting temperatures ranging from 40.5 to 82.0 °C in 150 mM potassium concentration.

### Strand-asymmetric PQS distribution is common but not universal in the Jockey clade

In order to find out whether a high number and asymmetric PQS distribution is specific for the HTT elements, we performed a comprehensive analysis of PQS distribution in the repetitive DNA of the *D. melanogaster* genome (dm6; https://www.ncbi.nlm.nih.gov/assembly/GCF_000001215.4/). Using RepeatMasker (version 4.1.1; http://www.repeatmasker.org), the internal *D. melanogaster* specific repeat database was employed for repetitive DNA identification. Subsequently, the PQS were searched in masked and repetitive genomic fractions by the pqsfinder software. We found that the repeats (representing 18.4% of the genome) contained a proportional number of PQS (17.2% of all PQS), suggesting that repeats are not PQS enriched compared to the rest of the genome (Additional file 1: Fig. S2).

**Fig. 2:**
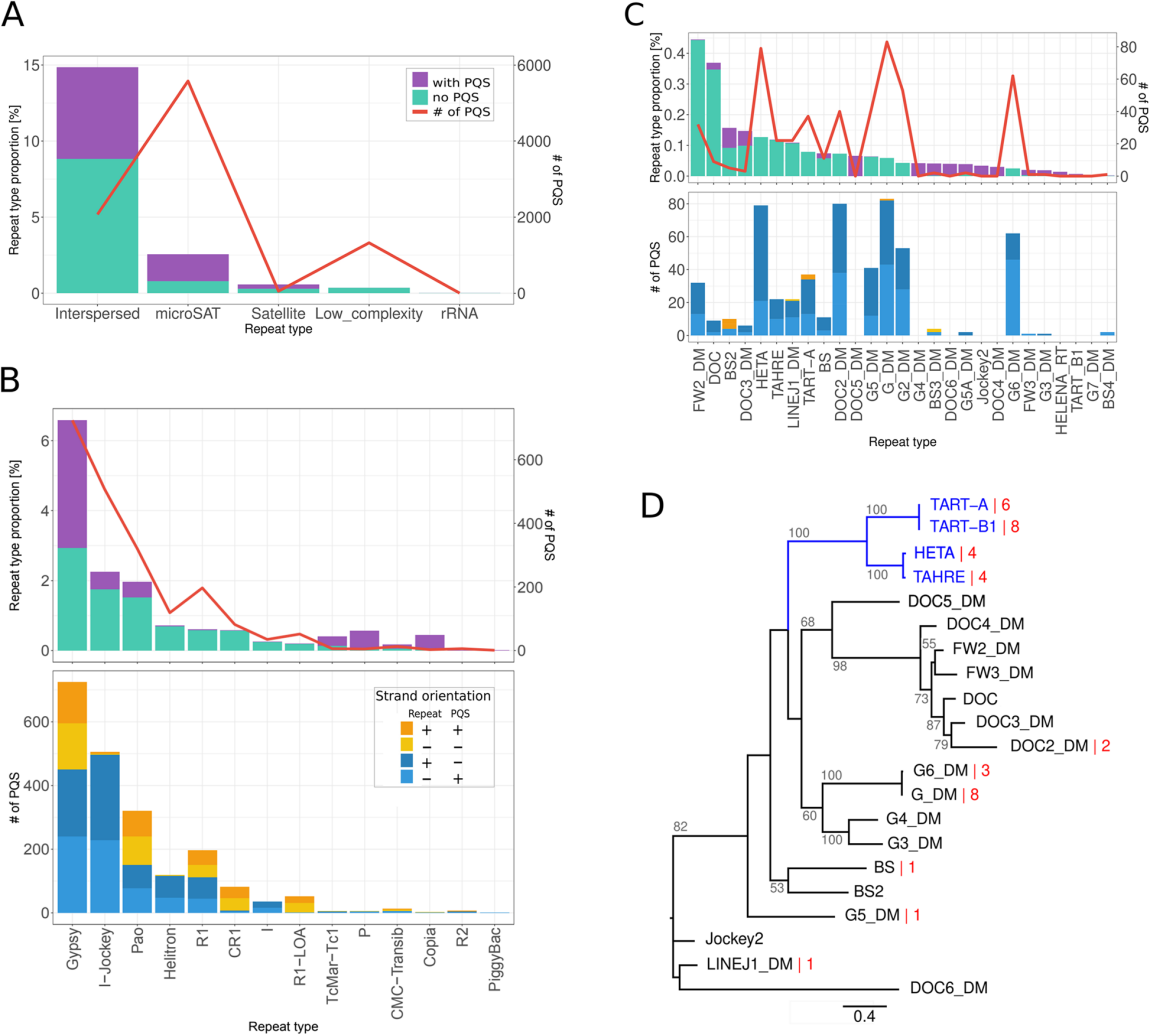
Identification of main PQS carriers among repetitive DNA in *D. melanogaster* genome. **A** Genomic proportion and PQS content of 5 main repeat types. **B** Analysis of the main interspersed repeats. Top: genomic proportion and PQS counts in individual types of interspersed elements. Bottom: mutual orientation of individual types of interspersed elements in the genome with respect to PQS orientation. The orange/yellow colors represent elements with sense PQS orientation—the G-rich sequence is on the coding strand, the blue colors represent elements with antisense PQS orientation (the repeat orientation is with respect to the chromosome, the orientation of PQS is with respect to the element in which PQS resides). **C** Analysis (as in **B**) of HTT harboring Jockey-clade elements. **D** GAG protein domain based phylogram shows the PQS distribution per reference full-length Jockey elements (red numbers, Additional file 2: Tab. S3). Note that G2 is not included due to the lack of GAG domain. Maximum likelihood phylogenetic tree was constructed using PhyML v3.0 with BioNJ used to build up the starting tree. Bootstrap support values higher than 50 are given

To get an general idea about the PQS distribution among various repeat types we looked at repeats in detail (Fig. 2). Interspersed repeats constitute almost 15% of *D. melanogaster* genome and harbor approximately 20% of all PQS originated from repetitive DNA. On the contrary, rather short sequences including microsatellites and low complexity repeats less abundant in the genome represent the main PQS pool among repeats.

The detailed analysis of the most abundant transposable elements showed that Jockey elements (subclade of LINE elements harboring HTTs) together with Gypsy and Pao elements (LTR retrotransposons), were the most PQS rich transposons (Fig. 2B). A remarkable fact was that Jockey elements, together with Helitrons and closely related I-elements, exhibited a strict antisense PQS orientation which was in contrast to other predominant transposable elements, namely Gypsy and Pao families (Fig. 2).

A closer view at the PQS density within Jockey clade families showed that the relatively high PQS frequency was not limited to telomeric HTTs, but also to some other families (e.g. DOC2, G, G2, G5 and G6; Fig. 2C). However, representatives of some Jockey families showed no or low G4-forming potential (e.g. DOC, DOC5, G4, BS3 and others). Because most of the Jockey elements are fragmented, we further analyzed those Jockey fragments containing PQS and normalized them by PQS size in base pairs to the length of respective fragments (Additional file 1: Fig S3). The highest PQS proportion was recorded in BS elements (19.3 and 22.2% for BS3 and BS4, respectively), whereas the HTT elements revealed values comparable with those for groups of G, DOC and FW elements (i.e. 5.0, 4.2 and 1.6% of PQS in Het-A, TART-A and TAHRE, respectively; Additional file 1: Fig S3). The phylogenetic analysis of Jockey full-length reference element sequences uncovered a scattered PQS distribution in non-telomeric families whereas the HTTs (clustered together in monophyletic clade) carried four to eight PQS (Fig. 2D).

### Asymmetric PQS distribution is common for telomeric elements in other *Drosophila* species

**Fig. 3:**
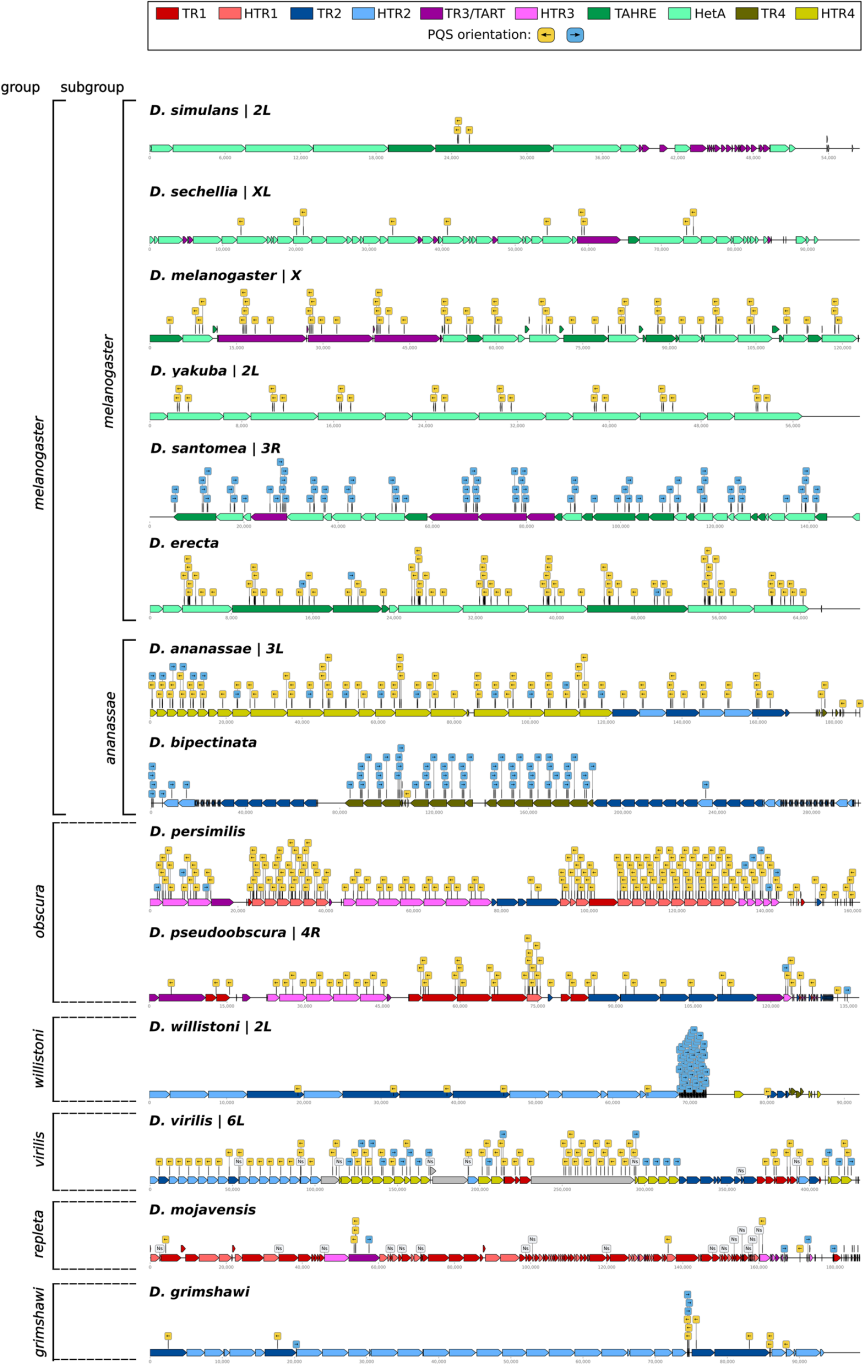
Visualization of LINE retrotransposons arrays with PQS in telomeric sequences of various *Drosophila* species. Mutual strand-specific orientation of LINEs and PQS is depicted. The order and taxonomic division of given species was adapted from the *Drosophila* genus consensus phylogenetic tree in [24]. The gray colored boxes in *D. virilis* are formed by tandem repeats TTR321 and TTR712. The PQS rich region in *D. willistoni* is formed by 392 bp long tandem repeat. Note that in each species there is at least one element with antisense PQS

In order to determine how common the phenomenon of strand asymmetric PQS distribution is in telomeric retrotransposons, we analyzed the PQS abundance and orientation in telomeric elements from 13 other species of *Drosophila* genus (Fig. 3). Based on previously published telomeric transposons [10, 22] we created the largest comprehensive database of reference full-length telomeric transposons up to date (Additional file 3, for details see Methods). However, as reported previously [22], some species (e.g. *D. ananassae*) contain a large number of lineages of particular elements which are not covered by our database. In addition to the previously reported tandem repeats involved in telomere maintenance in *D. virilis* [23] we found 3 new tandem repeats associated with telomeric transposons. Based on monomer length we named these telomeric tandem repeats TTR321, TTR712 and TTR2388. All three contain poly(A) tracts in the same orientation as the telomeric transposons suggesting that they possibly originate from an array of transposons.

Based on RT phylogeny, the telomeric elements can be divided into three groups; TR1, TR2, and a large TR3 group (Additional file 1: Fig. S4A) [22]. The TR3 group could be further subdivided into subgroup TR3/TART, well supported TAHRE subgroup and TR4 subgroup that is quite divergent and clusters differently based on the GAG protein. TR elements that lost the *pol* and contain only the *gag* gene were called half-transposable elements (HTR) and based on the *gag* similarity belong to the respective TR group, similarly as Het-A and TAHRE. The HTR can be found in every group except for TART. While the TR2 group showed the lowest amount of PQS per element comparable to non-telomeric *D. melanogaster* Jockey elements, all the other groups showed higher PQS counts, where TR3 and TAHRE had the highest values (Additional file 1: Fig. S4B).

In agreement with our findings in *D. melanogaster* we found at least one telomeric element that contained PQS in an antisense orientation in all analyzed *Drosophila* species (in relation to element genes; Fig. 3). However, in most species we observed that the PQS abundance in telomeric transposons was much higher with regards to both the PQS per element and the number of elements with antisense PQS. The PQS density in telomeric arrays showed that *D. virilis*, *D. mojavensis, D. willistoni* and *D. grimshawi* contained less PQS and were predominantly occupied by PQS-poor TR1 and TR2 elements (Fig. 3, Additional file 1: Fig. S5).

The *melanogaster* subgroup (dominated by TAHRE and TR3/TART elements) contained elements with a higher PQS density, except for the phylogenetically sibling species *D. simulans* and *D. sechellia*. The *ananassae* subgroup contained a moderate PQS density and was occupied by TR2 and TR4 elements. Interestingly, the species forming the *obscura* group (*D. persimilis* and *D. pseudoobscura*) showed one of the highest PQS densities in telomeric transposon arrays that were formed by the combination of TR1, TR2 and TR3 elements (Additional file 1: Fig. S5). In addition, we detected PQS in SAR-repeats and Helitron transposons composed telomeres of *D. biarmipes* which show similar PQS density with telomeres of *D. bipectinata* and *D. virilis* (2.02, 1.73 and 2.03 PQS per 10 Kbp, respectively).

### PQS rich regions in *Drosophila* telomeric retrotransposons show higher DNA conservation favoring G4 formation and overriding amino acid conservation

**Fig. 4:**
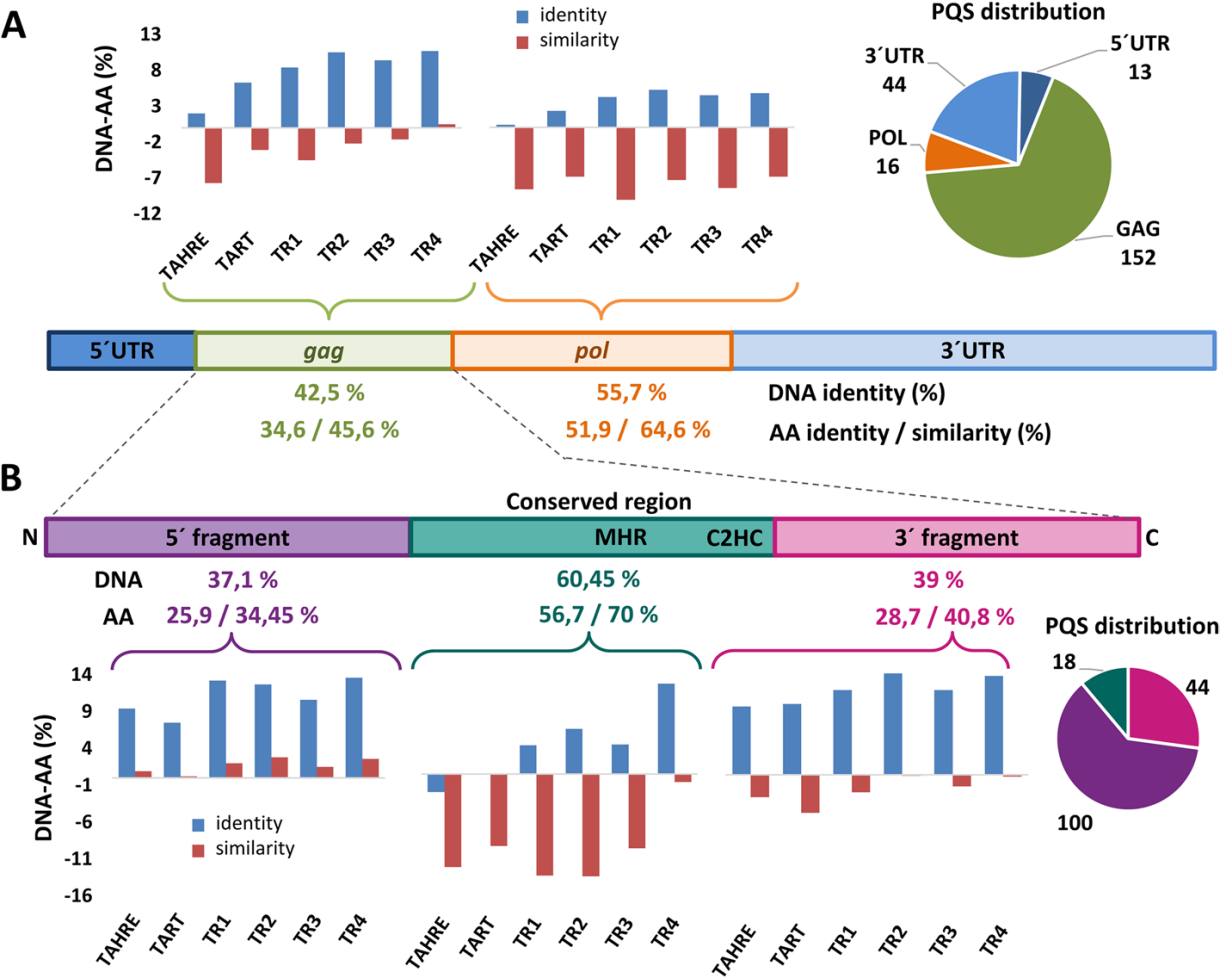
Analysis of DNA-AA identity bias with respect to PQS distribution. **A** The scheme of non-LTR retrotransposon shows the average pairwise DNA and AA identity or similarity calculated as an average of each group with respect to functional regions of non-LTR retrotransposon. The charts above them show the difference of average DNA-AA identity and similarity as defined by BLOSUM62 for *gag* and *pol* genes for different telomeric retrotransposon groups. The distribution of PQS in functional regions is indicated by a pie chart. **B** Dissection of DNA-AA identity and similarity in *gag* gene that can be divided based on protein alignment to conserved area and the rest that shows little conserved motifs. The *gag* is divided by the conserved region to 5´fragment (N-terminal) and 3´fragment (see Additional file 1: Fig. S6; C-terminal; major homology region—MHR; zinc knuckle motifs—C2HC). As for **A**) the average DNA and AA pairwise similarities and identities derived from all elements are shown for corresponding regions as well as the DNA-AA difference for the transposon groups. The pie chart shows PQS distribution in the 3 gag regions, note that 5 PQS are on the border of the conserved region and the 5´fragment creating a difference of 10 between gag located PQS **A**) and sum of PQS in gag regions **B**). The difference is 10 since these 5 PQS were counted for both regions

Casacuberta et al*.* showed that telomeric elements from four distant Drosophila species exhibit higher DNA identity compared to amino acid (AA) identity [25]. Consequently, they suggested that the primary DNA sequence and/or some structures may be important for the elements or telomere homeostasis. If we could correlate higher DNA conservation with G4-forming potential, such analysis would support the importance of G4 structures. Having a comprehensive database from 14 *Drosophila* species containing 84 telomeric elements we were able to inspect and validate the phenomenon of DNA-AA identity bias on a much larger scale. Moreover, we also took into account chemical properties of AA reflected by BLOSUM62 substitution matrix (AA similarity). We used a MUSCLE algorithm [26] to align the GAG and POL protein coding sequences at both DNA and AA levels and compared the average DNA-AA identity and similarity difference (Fig. 4A). In order to have as homologous elements as possible, we further separated the TART elements from the TR3 group for this analysis, since TART elements were specific for the *melanogaster* subgroup and hence also phylogenetically clustered.

We found that DNA-AA identity bias was common for all the telomeric retrotransposons and *Drosophila* subgroup Jockey elements (Fig. 4A, Additional file 1: Fig S7). In addition we inspected representatives of other LINE clades discussed below (R1, L1-like, CRE) which also revealed DNA-AA identity bias. However, when AA similarity was used AA showed higher conservation compared to DNA. This suggests that the parameter of AA identity favors DNA which is, however, not true for genes coding histone H3 and alcohol dehydrogenase that showed higher AA identity (Additional file 1: Fig. S8).

Nevertheless, the DNA-AA difference was much higher for *gag* gene compared to *pol*. Since the *gag* gene also harbors most of the PQS, we looked at the *gag* gene further in detail to see if there are certain regions behaving differently (Fig. 4B). Based on the AA alignment the GAG protein can be divided into three parts: central conserved region (AA 468–685 for Dmel_Het-A) and N-/C-terminal regions that showed only a little conservation (Additional file 1: Fig. S6). These 3 regions also differ in PQS distribution. While the conserved region is PQS poor, the N-/C-terminal regions are PQS rich where most of the PQS are located in the N-terminal region (Fig. 4B, Additional file 1: Fig. S6A). Interestingly, the 5´ region also showed higher DNA conservation even when AA similarity was used.

To have a control for regional differences of DNA/AA similarity in the *gag* gene, we gathered J1 elements from the *melanogaster* subgroup. We were able to retrieve intact J1 elements only from *D. simulans, D. sechellia, D. melanogaster and D. yakuba* and hence we tested HTT elements only from these species. We identified the regions in J1 *gag* genes based on the similarity with Dmel_Het-A. Both TART and TAHRE/Het-A showed higher DNA conservation in 5´ region compared to AA similarity while non-telomeric J1 did not (Additional file 1: Fig. S8). More detailed analysis of GAG coding regions showed that (i) dN/dS ratio revealed more frequent nonsynonymous substitutions in GAG and in 5´ region in particular (Additional file 1: Fig. S9), (ii) PQS loci prefer GC rich codons when compared with overall GC contents in respective sets of synonymous codons (Additional file 1: Fig S10), (iii) the cytosine usage probability in third codon positions is doubled and frequency of GC rich proline coded codons (GC contents 83.3%) is three (TART) to four (HeT-A and TAHRE) times higher in PQS loci (Additional file 1: Fig S11).

In other words, the region with the highest PQS abundance showed higher DNA conservation overriding AA conservation. This is supplemented by a higher frequency of nonsynonymous AA substitutions for AA with different physicochemical properties. Moreover, the PQS loci show preference for GC rich codons with higher preference for C at third codon position both of which would favor G4 forming potential.

### Telomere-associated retrotransposons show G4-forming potential in various species outside *Drosophilidae*

**Fig. 5:**
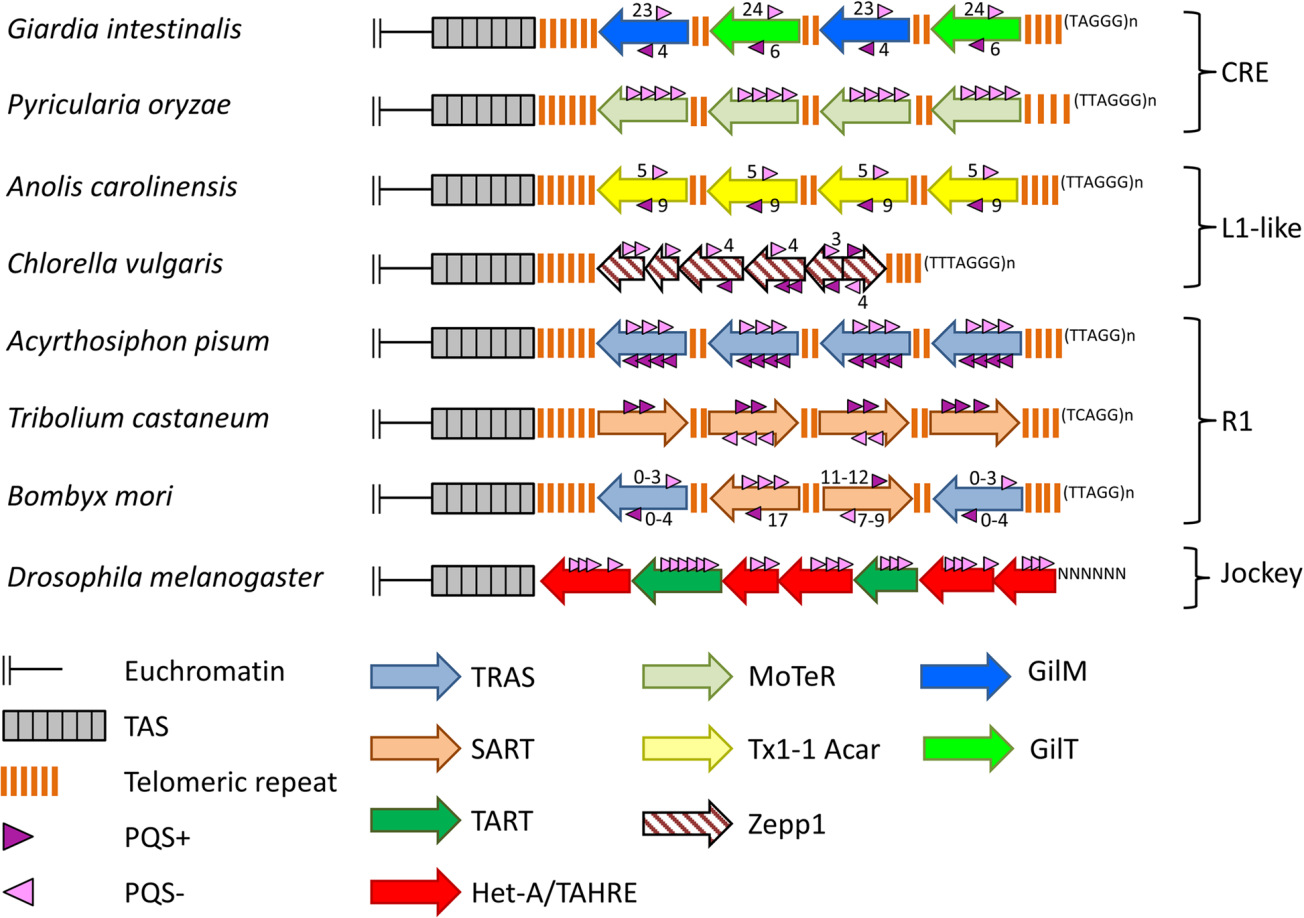
Telomeres with intercalated transposable elements containing PQS. Schematic representation of telomeric sequence structure in various species in which specific retrotransposons insert into canonical short telomeric repeats. All the elements belong to 4 LINE clades (indicated on the right). The PQS orientation is in relation to the respective element (PQS + means that the G-rich sequence is on the coding strand). The sequences in parenthesis represent both the telomerase-synthesized short tandem repeats as well as the terminal 3´ ssDNA overhang. TAS stands for telomere associated sequences

As shown above, we have found some evidence for G4 importance in *Drosophila* telomeric retrotransposons. However, the concern remains that the *D. melanogaster* HTT elements are only slightly enriched for PQS compared to the rest of the genome (4.15 and 4.47 PQS/10kbp in telomeres of 4 and X chromosome, respectively, *versus* 3.81 PQS/10kbp in whole *D. melanogaster* genome). Moreover, HTT elements show similar PQS content as some other Jockey elements in the genome. Hence we searched also for PQS in telomere-located elements in various eukaryotic species where telomeres are composed of both retrotransposons and short telomerase-synthesized repeats (Fig. 5). A finding of PQS in these retrotransposons would further support the importance of G4 in non-canonical telomeres. Although the majority of telomere-associated transposons are documented in insects (SART/TRAS elements—*B. mori, T. castaneum* and *Acyrthosiphon pisum* [1–3, 27]), other examples were also found in *Viridiplantae* (Zepp1—*C. vulgaris*) [5], Vertebrates (Tx1_Acar—*Anolis carolinensis*) [7], Fungi (MoTeR1—*P. oryzae*) [6], rotifers (Athena—*Adineta vaga*, *Phillodina roseola*) [28] and *Diplomonadida* (GilM and GilT—*G. intestinalis*) [4].

Surprisingly, we found that in most species these elements indeed showed high PQS density and often also strand-asymmetric distribution (Fig. 5). The only exception were Penelope-like Athena elements from bdelloid rotifers that lacked G4-forming potential except for telomeric repeats that are embedded in the elements [28]. GilM and GilT elements from *G. intestinalis* showed the highest PQS density containing 27 and 30 PQS respectively, most of which were on the same strand as G-rich ssDNA overhang. Even higher PQS strand asymmetry showed MoTeR1, having all G4 motifs in the same orientation as G-rich overhang (Fig. 5). In *T. castaneum*, the number and orientation of PQS differed based on the SART lineage between 0 to 5 per element, while most PQS were located in the same orientation as the G-rich overhang. In *B. mori* the telomeric regions were invaded by several lineages of two R1 retrotransposon families SART and TRAS that insert into telomeric repeat in opposite orientation (see Methods; [1, 29]). The number of PQS per element differed based on the lineage between 0–5 for TRAS elements and 18–21 for SART (for relevant sequences see Additional file 2: Tab. S1). Due to the high diversity of *B. mori* telomeric elements it was hard to assess which PQS orientation was dominant. The orientation of PQS in other species was more or less symmetrical in relation to the telomeric retrotransposons and/or G-rich overhang.

**Fig. 6:**
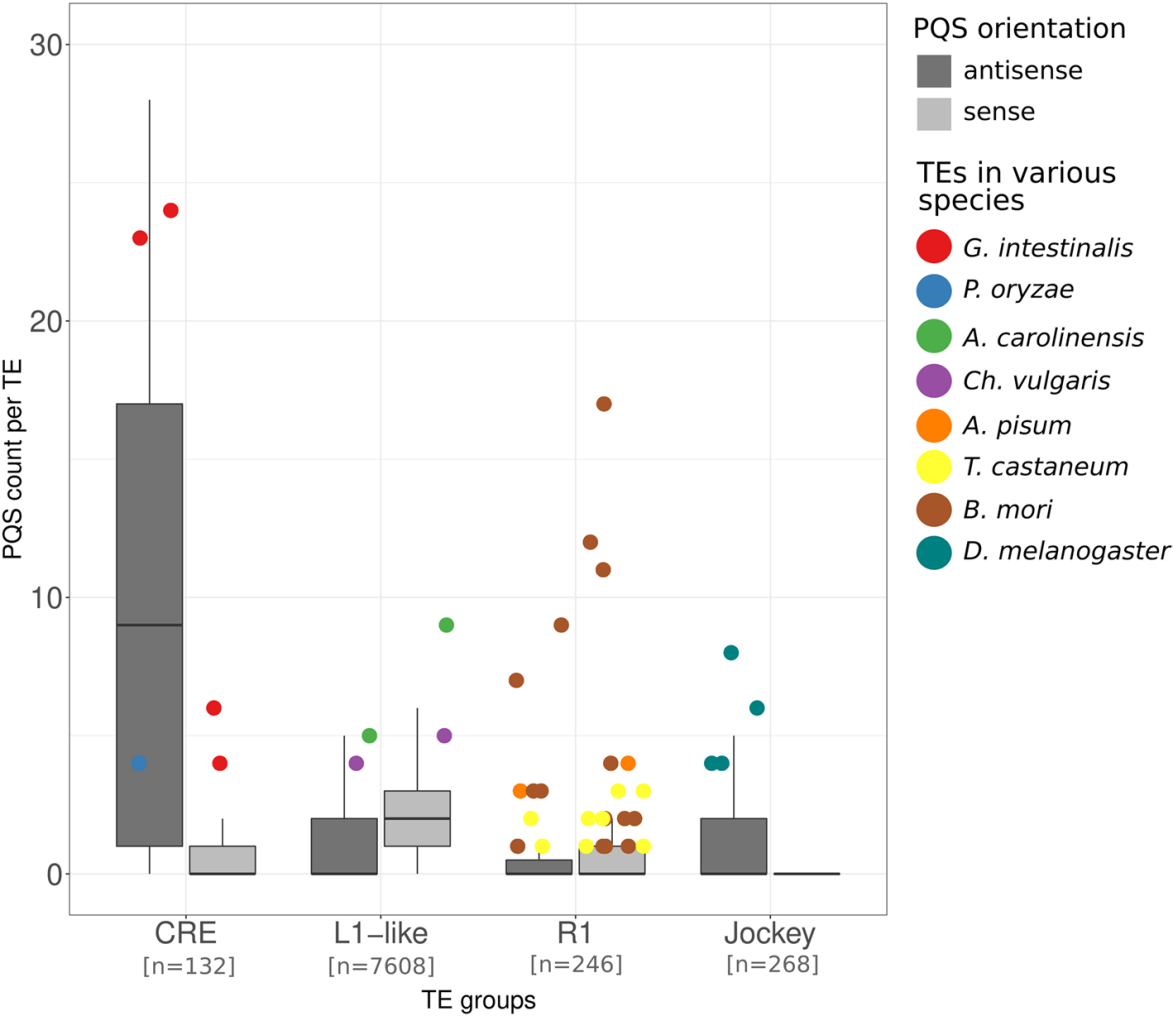
PQS counts in selected families of LINEs with the telomeric elements. Mutual strand-specific orientation of PQS and TE in 4 groups of LINEs showed in dark- and light gray for antisense and sense, respectively. PQS in telomeric elements of all species (stated in Fig. 5) are highlighted and tend to be the most PQS-rich elements in each group. Number of analyzed elements for each TE group is given

As described previously, all mentioned telomere-located elements belong to the four distinct LINE clades defined by the Dfam database: CRE, L1-like, R1 and Jockey (Fig. 5) [30]. The only two elements that were not assigned to particular LINE clade were GilM and GilT elements. Based on the RT-domain, these two elements are part of the CRE clade (Additional file 1: Fig. S12). To elucidate whether the telomere-located elements are enriched for PQS we gathered full-length elements from Dfam and Repbase (Fig. 6) and tested whether the PQS numbers in telomeric elements belong to the upper outliers within each LINE clade (Additional file 2: Tab.S2). Indeed, we found that all telomeric elements from Jockey and R1 clades and those with PQS in sense orientation in the CRE clade reveal a significantly higher G4-forming potential than the majority of their non-telomeric relatives.

## Discussion

The HTT elements that maintain the chromosome ends in *D. melanogaster* preserve the strand composition bias making one strand G-rich similarly to canonical telomeres [14]. However, this bias is subtle compared to canonical telomeres and its significance is not known. Here we documented the accumulation of strand-asymmetric PQS in HTT elements in *D. melanogaster* and verified that most of the PQS are able to form G4 in vitro*.* Our finding is supported by the reported presence of G4-signals on polytene chromosomes termini using 1H6 antibody [31, 32].

Our results suggest that retrotransposons could be selected for telomere protection thanks to the combination of unidirectional insertions and the strand-asymmetric G4-forming potential. The asymmetric PQS distribution in *D. melanogaster* Jockey clade, Helitrons and I elements makes these transposons a good candidate as documented by *D. biarmpies* where Helitrons are the major component of telomeres [10]. So far we can just speculate why the HTTs were evolutionarily adapted as telomere components instead of other PQS-rich Jockey elements (especially families G, G2 and G5). Perhaps PQS capacity was just one of the factors for their selection. Nevertheless, the uneven PQS distribution in *D. melanogaster* Jockey phylogenic tree, common occurrence of antisense PQS in telomeric retrotransposons of other *Drosophila* species and selection of PQS-rich elements in non-*Drosophila* species support the preference of PQS containing elements at telomeres.

The selection of PQS-rich elements for telomere maintenance seems to be of a secondary importance compared to the primary prerequisite of telomeric elements—an ability to target telomeres. All LINE elements may, in theory, use an existing 3´OH group to prime reverse transcription. This could be documented by the human L1 element that may take over the telomere elongation function if the telomere is dysfunctional [33]. Interestingly, the L1 element contains two G4-forming motifs [34].

The possible function of G4 in *Drosophila* telomeric retrotransposons (or telomeric retrotransposons in general) is hard to assess because, unlike in canonical telomeres, the G4 may be important for both the life-cycle of the retrotransposons themselves and/or for telomere capping or elongation. These two roles of G4 are difficult to distinguish since they are interconnected due to the fact that elongation happens via retrotransposon activity. Moreover, the function of G4 even in canonical telomeres is yet to be fully understood (reviewed in [13]). The function of G4 in transposable elements had been poorly studied and so there is only a limited amount of research data available. For instance, it has been shown that G4 located in the 3´UTR of L1 retrotransposons stimulates retrotransposition [35] and that G4 may affect both transcription and reverse transcription of Ty1 retrotransposon in yeast [36]. Here we have also found PQSs in two regions of the 3´UTR of *B. mori* SART1 elements which were reported to be essential for retrotransposition [37]. Interestingly, in the case of *B. mori* where the telomeric repeat does not readily form G4 [12], the telomeric retrotransposons may represent a donor of G4 structure.

Assumption that G4-formation stands behind the telomeric strand composition bias in the *Drosophila*, could point to the common functions of G4 shared with canonical telomeres. First, we should note that, compared to canonical telomeres, the G4/PQS density in the *Drosophila* species is much lower. However, it is still not clear how many and if any G4 are truly necessary even for canonical telomere capping or elongation (for review see [13]). It was shown on HTT deficient telomeres that the HTT elements are neither necessary nor sufficient for establishing a protective cap at the telomeres (for review see [38]). Nevertheless, as we showed, the PQS density in HTT arrays differs only slightly from the genome average. Thus, it is possible that some G4 would be preserved even at telomeres lacking HTT elements. Hence the importance of G4 for capping can not be excluded. On the other hand, such telomeres presumably lost the elongation function and shorten every fly generation, which only reflects that the telomeres are elongated via retrotransposition. Since Het-A GAG expression and the highest in vivo G4 formation are coupled to the S-phase in which telomere elongation takes place [39–41], G4 related function could be advantagous for telomere elongation in *Drosophila*.

The position and orientation of the G4-sequences in the *Drosophila* telomeric elements are somewhat peculiar. Firstly, the majority of the PQS were located in the coding region of the *gag* gene while in genomes the PQS are mainly accumulated in regulatory regions such as promoters or UTRs and are mostly excluded from coding regions [42, 43]. Secondly, in contrast to the above mentioned L1 and SART1, as a result of the PQS orientation, the G4 in HTT elements were not formed on a sense transcript. This implies a G4 function either on the DNA level or after the first cDNA strand synthesis. Nevertheless, all the HTT elements in *D. melanogaster* also produce antisense transcripts that are important for epigenetic maintenance of the telomeric HTT array and are more abundant in mutant stocks with long telomeres [44–47]. Moreover, the HTT arrays serve as both piRNAs clusters and targets for gene silencing where the antisense transcript is the piRNA precursor (reviewed in [48]). Several studies showed that G4s are implicated in piRNA biogenesis and activity modulation [49–51]. Besides, the antisense transcripts resemble telomeric repeat-containing RNA (TERRA). The G4 in TERRA have been shown to be crucial for interaction with TRF2 and hence telomere integrity [52]. The TERRA G4 have also been proven to mediate interaction with HP1α and the enrichment of this protein at telomeres [53]. Interestingly, the knockdown of HP1α in *D. melanogaster* leads to specific upregulation of HTT elements [54–58].

The contribution of G4 to telomeric retrotransposons and/or telomere maintenance in *Drosophila* sp. is further supported by the DNA-AA identity and similarity bias which was more pronounced in regions with higher PQS occurrence where nonsynonymous AA substitutions are more frequent and AA vary in physicochemical properties. We also showed that PQS loci are specified by usage of GC rich codon variants, higher frequency of cytosine in third codon position and proline coding codons. Proline-rich sequences in GAG domains could be a pointer to the retrotransposon life cycle regulation, since these areas are known to cause translational stalling [59]. This finding suggests that G4 may be the driving force for DNA sequence conservation or vice versa that some secondary DNA structures important for the elements may lead to the evolution of G4 conformation favorable for telomere maintenance. It is possible that once a hypothetical “G4 threshold” is reached under certain conditions (too great number, too pronounced stability, unfavorable topologies or combination of all), a recombination-based alternative telomere lengthening mechanism could be triggered [60].

Such a mechanism could eventually be represented by the formation of complex tandem repeats as in mosquitos [61]. A head-to-tail array of transposons as found in *Drosophila* sp. could be an ideal substrate for the formation of these repeats [62]. An acquisition of different transposons is also possible. *D. virilis* telomeres seem to represent a chimera of both tandem repeats probably originating from telomeric transposons and transposons themselves.

## Conclusions

Our results have brought a deeper insight into the nature of the non-canonical telomeres, namely the potential role of G4 in telomeric retrotransposons. Although we have not deciphered the function of G4 residing in telomere-associated retrotransposons, our findings of strand-biased G4-forming potential in multiple eukaryotic species support the importance of these structures in non-canonical telomeres. These findings call for further experimental validation of both in vivo formation of G4 in telomeric retrotransposons as well as the potential functions of these structures.

## Methods

### In vitro measurements

Circular dichroism (CD) spectroscopy and PAGE were performed as described in [63]. The CD measurements were performed at 23 °C. UV absorption spectroscopy and thermal melting assay were performed as described in [36]. The sequences of the oligonucleotides used are available in Additional file 1: Fig. S1B.

### *D. melanogaster* genome analysis

The *D. melanogaster* genome (dm6; https://www.ncbi.nlm.nih.gov/assembly/GCF_000001215.4/) was subjected to: (i) repetitive DNA analysis using RepeatMasker (version 4.1.1; http://www.repeatmasker.org) with internal *D. melanogaster* species specific repeat database and (ii) PQS (potential quadruplex-forming sequences) identification using pqsfinder software [17]. Outputs from both of the tools were formatted into GFF files (Additional file 4, 5) and the numbers of PQS in each respective repetitive DNA class was read from their intersections given by the bedtools package [64]. These datasets were used as default for the generation of Fig. 2 and Additional file 1: Fig S3. Moreover, in order to reveal the intraspecific variability in PQS capacity the pqsfinder was also employed for PQS detection in the consensus sequences of 5 Het-A subfamilies published by McGurk et al. (2021) (Additional file 1: Fig S1F; [19]). DNA features viewer Python library [65] was utilized for the visualization of *Drosophila* species telomeres with telomeric repeats, PQS and their mutual strand-specific orientation in Fig. 3 The phylogenetic order of *Drosophila* species and their division into respective (sub)groups in Fig. 3 is adapted from [24].

### Identification of main PQS carriers among repetitive DNA in *D. melanogaster* genome

Full-length sequences of all (both HTT and non-HTT) *D. melanogaster* non-LTR retrotransposons of the Jockey family were filtered from RepeatMasker Libraries (Additional file 2: Tab. S3). PQS counts in full-length elements were identified by pqsfinder. The GAG domains were (i) predicted using blastx [66] and reference GAG domains (for geneBank IDs see Additional file 2: Tab. S3); and (ii) aligned using the MUSCLE v3.8.425 algorithm [26]. The phylogenetic tree was constructed using Seaview version 4.7 (PhyML v3.0 with BioNJ used to build up the starting tree Fig. 2D; [67]). The G2 Jockey element is missing this phylogram because of the absence of a detectable GAG domain within its sequence. The same approach for phylogenetic tree construction was also used for Additional file 1: Fig. S4A and Fig. S12. Sequences used for phylogenetic tree construction are available in Additional file 2: Tab. S4, S5 and S6 respectively.

### Identification of telomeric transposons in *Drosophila* species and telomeric scaffolds annotation.

To develop a comprehensive database of telomeric transposons we used sequences from [10, 22] to build a custom Repeat Masker database and as queries for BLAST (or tBLASTn) on whole genome contigs, since available sequences were often partial or protein domains. We searched for scaffolds/contigs with array of open reading frames in one orientation at the end (or chromosomal ends whenever possible) and using Repeat Masker for annotation combined with manual curation we were able to substantially enrich the database of reference full-length telomeric transposons (Additional file 3, Additional file 2: Tab. S7). Due to the lack of experimental validation of telomeric localization the telomeric origin of unplaced genomic scaffolds was only potential. Identification of tandem repeats in *D. virilis* was performed using Tandem Repeats Finder [68] and they are included in Additional file 3. Due to the high variability of some elements in assorted species (not covered in our database) the Repeat Masker returned fragmented annotations that were manually curated for the generation of Fig. 3, the accessions and ranges of visualized scaffolds are in Additional file 2: Tab. S8.

### Assessment of DNA and amino acid (AA) identity bias

To compare the DNA and AA identity (Fig. 4) we aligned the GAG and POL protein sequences using the MUSCLE v3.8.425 [26] algorithm in Geneious R8.1.9 (https://www.geneious.com). The GAG protein was further subdivided into three domains based on overall conservation (see Results for details) and the alignments were trimmed to respective domains. Based on GAG protein subdivision corresponding DNA sequences were analyzed in the same way. The DNA alignments were performed using alignment by translation. The average pairwise identity and AA similarity based on BLOSUM62 were used for calculations. All sequences used for this analysis are available in Additional file 2: Tab. S9 and S10. Both DNA and AA alignments of *gag* genes from telomeric retrotransposon are available in Additional file 6. PQS that were on the border of GAG domains were counted for both domains. The sequence logos were generated from all GAG proteins using Geneious R8.1.9. Sequences used for comparison of other LINE elements (Additional file 1: Fig. S7) were either obtained using BLAST or downloaded from Dfam [69] and RepBase [70] and are available in Additional file 2: Tab. S11. We used SNAP web based software to calculate the dn/ds ratios from multiple alignments and the values are averages [71]. All data for figures regarding DNA-AA conservation bias, dn/ds ratios and accessions to genes for histone H3 and alcohol dehydrogenase are available in Additional file 2: Tab. S12.

In order to obtain a deeper view into DNA – AA relationships, we conducted the subsequent analysis. The GAG coding sequences of representative retrotransposons were filtered and divided into three family groups – Het-A, TAHRE, TART. Thereafter, the related sequences were aligned using MUSCLE and then SNAP software [72] was employed to obtain all aligned codons and their corresponding amino acids. Python custom scripts were generated to filter: (i) GC content for each codon which was compared with GC content of synonymous codons for respective amino acid ([73]; see Additional file 1: Fig. S10); (ii) areas in GAG sequences with predicted PQS and those without PQS were compared for the cytosine proportion in third codon position and the proportion of amino sequences usage (Additional file 1: Fig S11).

### Analysis of telomere targeting retrotransposons

The sequences of telomere targeting elements in Fig. 5,6 and Additional file 1: Fig. S12 were obtained from original papers—references and accession numbers are included in Additional file 2: Table S1. Since the published TRAS sequences in *B. mori* were mostly partial [1], we searched the current genome assembly (Bmori_2016v1.0) using BLAST and subsequently RepeatMasker with previously described elements as a custom database. Several chromosomes were terminated by SART/TRAS arrays. Annotation using ORF prediction (Geneious) and manual curation showed that there were at least 2 other lineages of SART elements, one of which seemed to insert in an opposite orientation (SARTr).

## Supplementary Information

**Additional file 1: Figure S1.** Localisation of PQS with in reference HTT elements and in vitro G4 formation validation. **Figure S2.** Proportions of repetitive DNA and PQS in *D. melanogaster* genome. **Figure S3.** PQS proportion of Jockey clade elements in the genome of D. melanogaster. **Figure S4.** PQS abundance of telomeric retrotransposons in 14 Drosophila species. **Figure S5.** PQS density in telomeric retrotransposon arrays of the Drosophila genus. **Figure S6.** Sequence logos of conserved motives in GAG proteins from Drosophila species telomeric retrotransposons. **Figure S7.** LINE elements show higher DNA conservation only if AA identity is used. **Figure S8.** HTT elements show higher DNA conservation in gag 5´region compared to J1 Charts. **Figure S9.** 5´ region of the gag gene show the highest dn/ds ratio Charts. **Figure S10.** Delta GC content in GAG codons. **Figure S11.** Comparison of PQS and non-PQS regions within GAG coding sequences of Drosophila telomeric retrotransposons. **Figure S12.** RT-based phylogram of representative LINE elements showing that GilM/GilTelements belong to the CRE-like elements.

**Additional file 2: Table S1.** Sequences used for Fig. 5. The scheme is based on the sequences and description in corresponding references. **Table S2.** Evaluation of G-forming potential of telomeric LINE elements. **Table S3.** Sequences of D. melanogaster Jockey elements used for construction of Fig. 2D. **Table S4.** Sequences used for RT based phylogeneitc tree construction in Figure S4A. **Table S5.** Sequences used for GAG based phylogeneitc tree construction in Figure S4A. **Table S6.** RT domains used for construction of Figure S12. **Table S7.** Telomeric and potential telomeric scaffolds used for PQS density assesment (Figure S5). All Ns were removed from the analysis but are included in used regions. All regions that we could not indentify faithfully as telomeric elements were removed from analysis and are not included in used regions. **Table S8.** Sequences used for visualisation of telomeric and potential telomeric scaffolds in Fig. 3. The "Start & Stop" postions correspond to visualized regions. **Table S9.** GAG DNA and AA sequences used for Fig. 4. Conserved domain coresponds to bp 1402–2055 and AA 468–685 in Dmel_HetA. **Table S10.** Pol (RT) DNA and AA sequences used for Fig. 4. **Table S11.** Sequences used for Figure S7. Aminoacid sequences can be obtained by simple translation of these sequences. **Table S12.** DNA and AA identity and similarity, including dn/ds ratios.

**Additional file 3.**

**Additional file 4.**

**Additional file 5.**

**Additional file 6.**

## Data Availability

All data generated or analysed during this study are included in this published article and its supplementary information files. Raw data used for figure generation are available upon reasonable request.
