# Supplementary material for: Telomeric retrotransposons show propensity to form G-quadruplexes in various eukaryotic species
Source: Mob DNA. 2023 Apr 10;14:3. doi: 10.1186/s13100-023-00291-9 (PMC10088271; doi:10.1186/s13100-023-00291-9)
Supplement: Supplementary file 1 — Additional file 1: Figure S1. Localisation of PQS with in reference HTT elements and in vitro G4 formation validation. Figure S2. Proportions of repetitive DNA and PQS in D. melanogaster genome. Figure S3. PQS proportion of Jockey clade elements in the genome of D. melanogaster. Figure S4. PQS abundance of telomeric retrotransposons in 14 Drosophila species. Figure S5. PQS density in telomeric retrotransposon arrays of the Drosophila genus. Figure S6. Sequence logos of conserved motives in GAG proteins from Drosophila species telomeric retrotransposons. Figure S7. LINE elements show higher DNA conservation only if AA identity is used. Figure S8. HTT elements show higher DNA conservation in gag 5´region compared to J1 Charts. Figure S9. 5´ region of the gag gene show the highest dn/ds ratio Charts. Figure S10. Delta GC content in GAG codons. Figure S11. Comparison of PQS and non-PQS regions within GAG coding sequences of Drosophila telomeric retrotransposons. Figure S12. RT-based phylogram of representative LINE elements showing that GilM/GilTelements belong to the CRE-like elements. [file 13100_2023_291_MOESM1_ESM.pdf]

Figure S1. Localisation of PQS within reference HTT elements and *in vitro* G4 formation validation

A) Schematic localisation of potential quadruplex forming sequences in reference HTT elements predicted using pqsfinder. Note that PQS no 9 in TART originates from TART-A1 (AY561850) at the respective position. PQS with a red number does not form G4 *in vitro* **B**) Predicted PQS with 2 nt of surrounding sequences as used for *in vitro* measurements. All the sequences are reverse complements since they are present on template strands. Corresponding No. for Fig. 1 is indicated as well as the score from pqsfinder, G4 topology based on CD spectroscopy (Anti - antiparallel; Para - parallel; 3+1 - hybrid, NA - not applicable) and melting temperatures in 15mM K+ (Tm15) and 150mM K+ (Tm150). **C)** Alignment of reference elements from TART subfamilies shows different numbers of PQS (gray triangles). Each position containing PQS is depicted with sequence logo as well as translation of the *gag* (green arrow) and *pol* gene (purple arrow). UTRs are blue. **D)** Comparison of Het-A and TAHRE elements as in C) **E)** Sequences from (Abad and Villasante, 1999) maped to Het-A (U06920) note that the annotations are derived directly from Genebank. **F)** comparison of PQS found in consensus sequences of 5 Het-A subfamilies (McGruk et al, 2021) reflecting intraspecific variability. **G)** From top to bottom: Full CD spectra in increasing K+ concentration (ON - overnight incubation), melting curves expressed as normalized absorbance at 297 nm, melting curves at 297 nm expressed as excitation coefficient. Thermal stability was measured in 150mM K+.

A

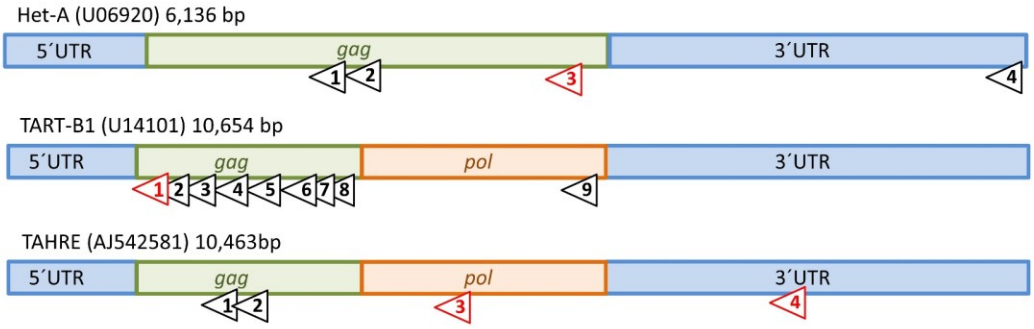

B

Element	PQS	Fig.1 No	Sequence	Score	G4	Tm15	Tm150
HetA	1	1	CAGGCGAGGGCCGGCGGGGGGGTC	66	Anti	38.5	60.5
	2	2	GAGGGGGTAGGGGGGCGATCCCGGGTG	64	Para	NA	82
	3		CAGGTGGCGGGGGTGGTTCTTGGTTTGGTGCAACAGGAA	47	NA	NA	NA
	4	3	ATGGGGTGAGTTTGGGGGTTGGGCGTGGTG	58	Para	29.5	55
TAHRE	1	1	CAGGGTAACGGCTGGCGGGGGGGTC	49	Anti	26	49.2
	2	2	GAGGGGGTAGGGGGGCAATCCCGGGTG	64	Para	NA	80
	3		GCGGTTATTTTGGGGGCGATTAGGTGGGGTGGCTTCATTGGCT	52	NA	NA	NA
	4		CTGGGGACCGCCCAAAGATTGGGCCATGGGAATGGGTG	49	NA	NA	NA
TART	1		ATGGGGAAGGGGCTATTGGCAGGCTTTTTTTCATTGGCTTCTGTGGTA	50	NA	NA	NA
	2	1	TTGGAGGGGGGCGAGGGTTTGGTGGGCT	64	Anti	25.5	53
	3	2	TTGGGTGCGGGGTGAGGGGTCTTTGGCGGGCT	64	Anti	37	58.5
	4	3	CTGGGAGAGGGGCGAGGGGCTTAAAGCGGGCT	63	Anti	32	51
	5	4	CTGGGCTTGGGTTTGTGTCTTGGGCGGGAT	58	Para	34	58
	6	5	GCGGGGCGTCTGAAGGTGGGTGGGTTTTGGGTAGGGATGTTTATTGCGGGGGGTC	71	Para	40.5	61.5
	7	6	GAGGGAGGGGGTGTGTGTAGGGCT	52	3+1	NA	40.5
	8	7	AAGGGGGTGGGCGGAAATGAGTGAGATGGGTGTTGGCCGGGTTTAGGAC	73	Para	NA	51.5
	9	8	TTGGGGAGGGGTGGTATATGTGTGGGAGTGGCA	54	3+1	NA	50.5

C

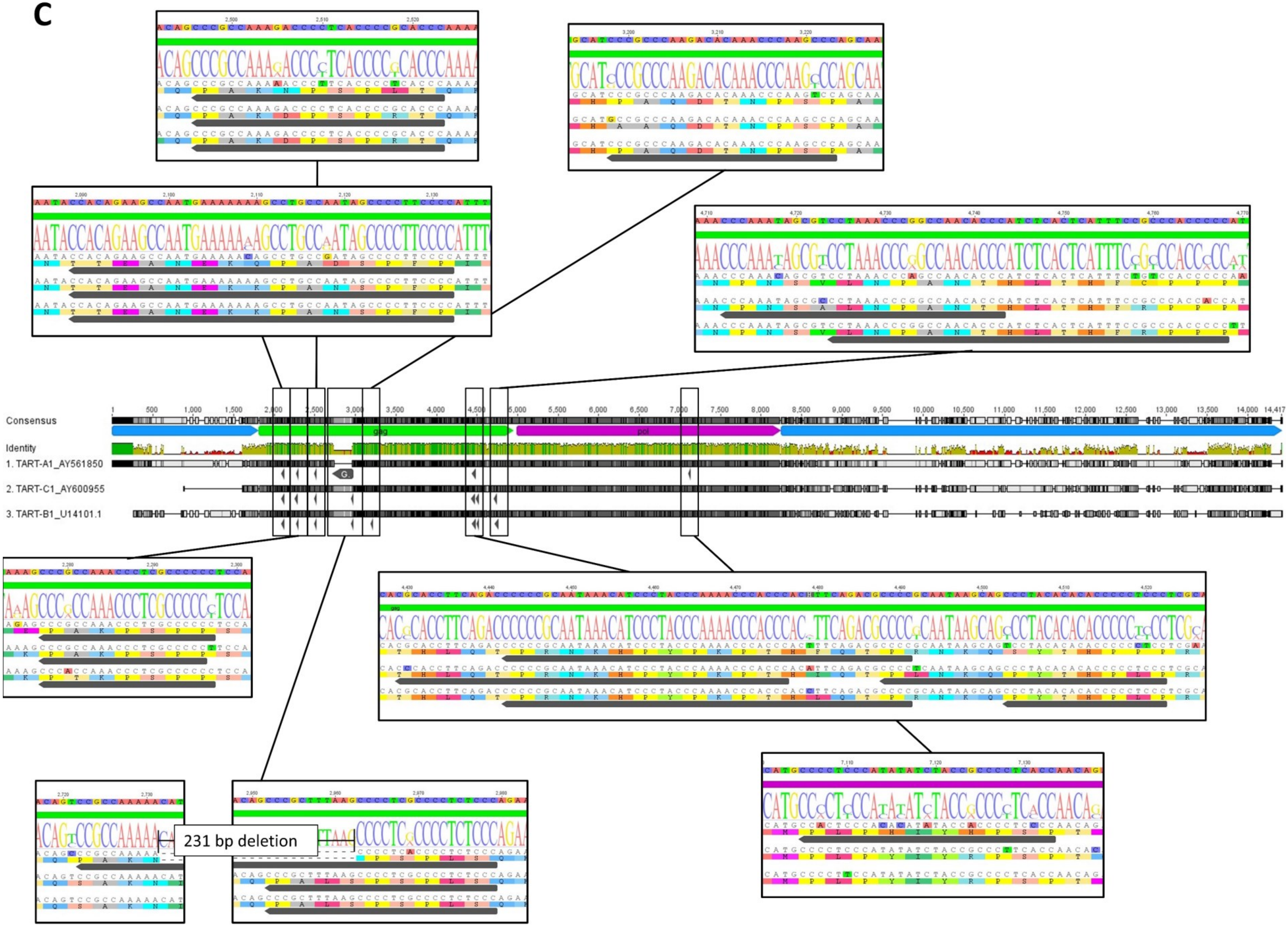
Consensus
Identity
1. TART-A1_AY561850
2. TART-C1_AY600955
3. TART-B1_U14101.1
gag
pol
231 bp deletion

D

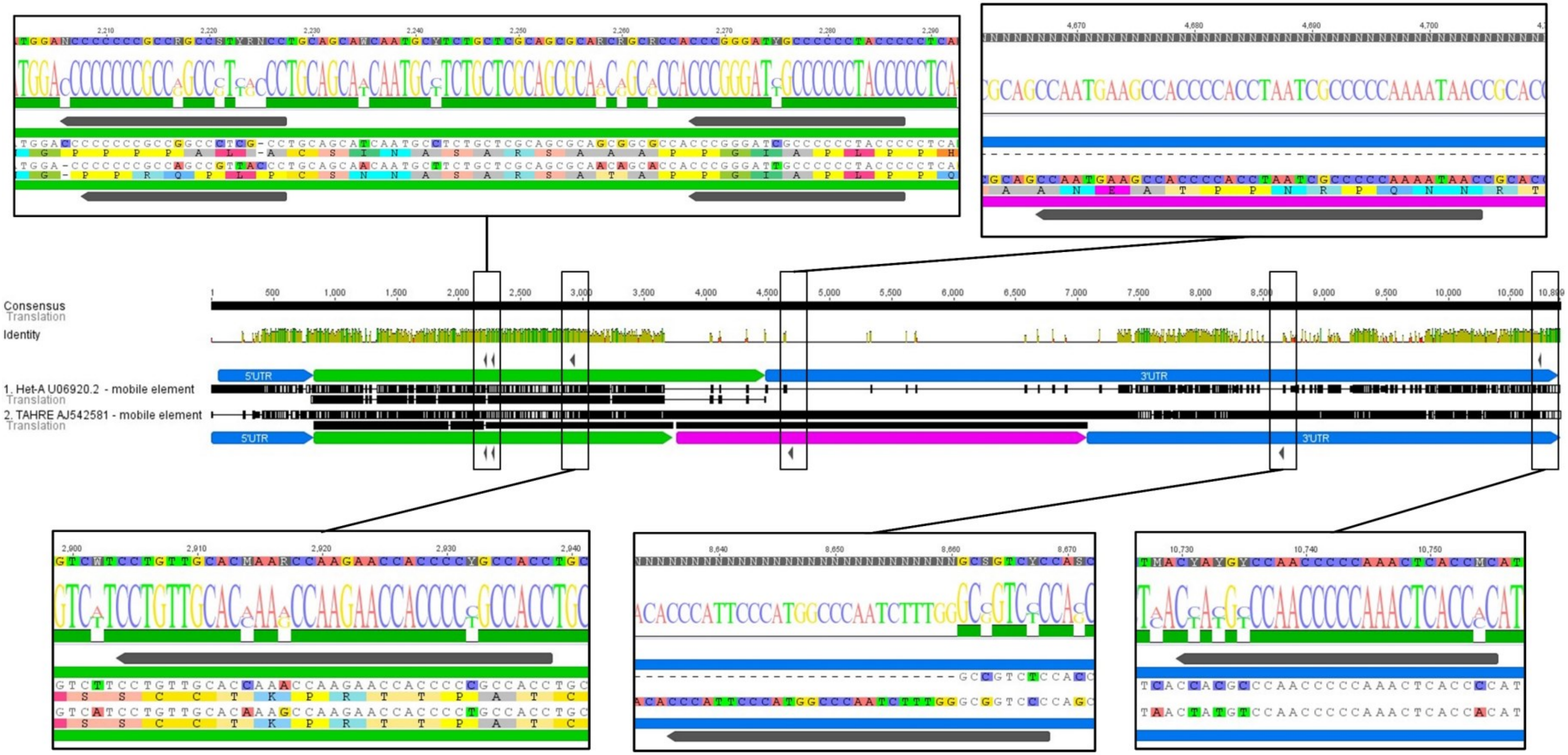

Consensus
Translation
Identity
5'UTR
3'UTR
1. Het-A U06920.2 - mobile element
Translation
2. TAHRE AJ542581 - mobile element
Translation

E

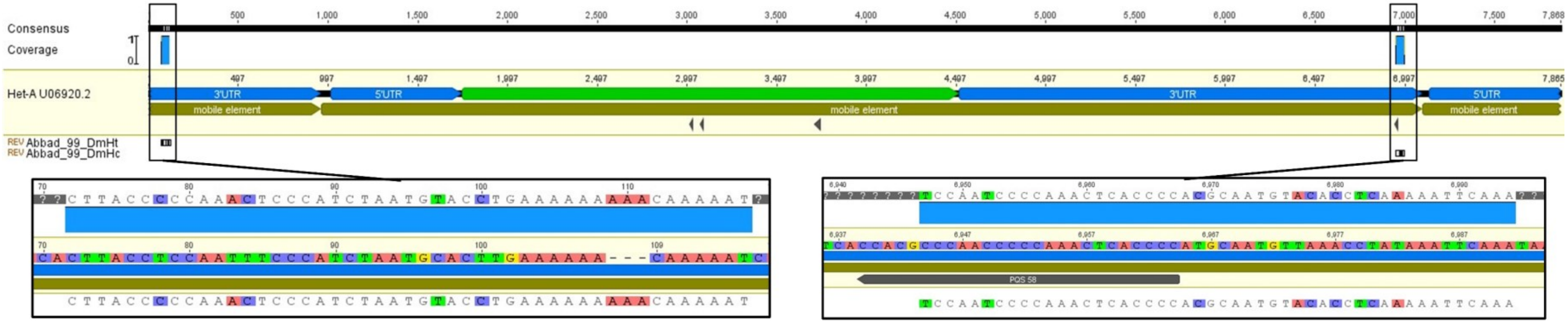

Consensus
Coverage
Het-A U06920.2
3'UTR
5'UTR
mobile element
REV Abbad_99_DmHt
REV Abbad_99_DmHc
PQS 58

F

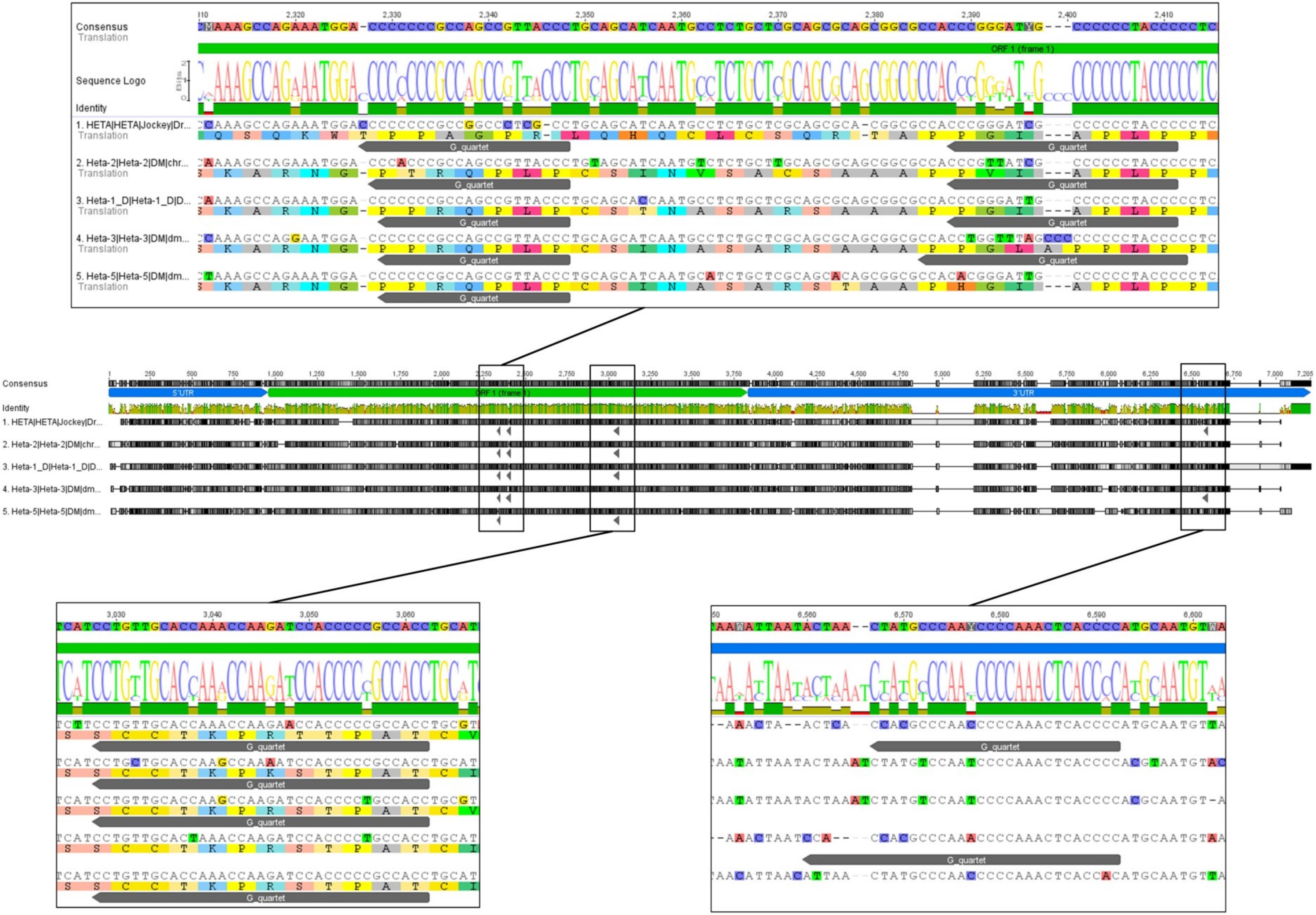

Consensus
Translation
Sequence Logo
Identity
1. HETA|HETA|Jockey|Dr...
2. Heta-2|Heta-2|DM|chr...
3. Heta-1_D|Heta-1_D|D...
4. Heta-3|Heta-3|DM|dm...
5. Heta-5|Heta-5|DM|dm...
ORF 1 (frame 1)
5'UTR
3'UTR
G_quartet

G

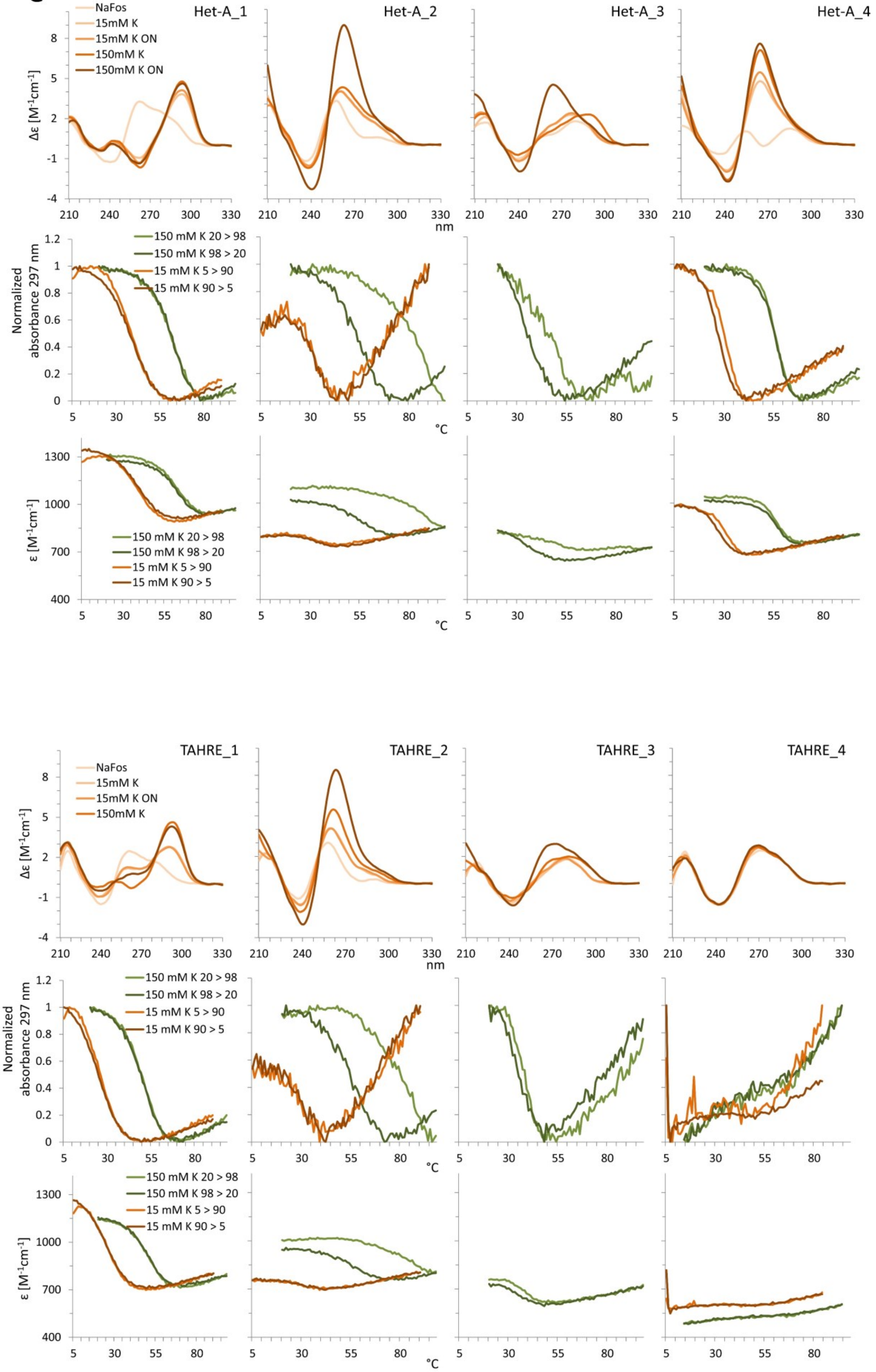
Het-A_1
Het-A_2
Het-A_3
Het-A_4
NaFos
15mM K
15mM K ON
150mM K
150mM K ON
Δε [M-1cm-1]
nm
Normalized absorbance 297 nm
150 mM K 20 > 98
150 mM K 98 > 20
15 mM K 5 > 90
15 mM K 90 > 5
°C
ε [M-1cm-1]
TAHRE_1
TAHRE_2
TAHRE_3
TAHRE_4

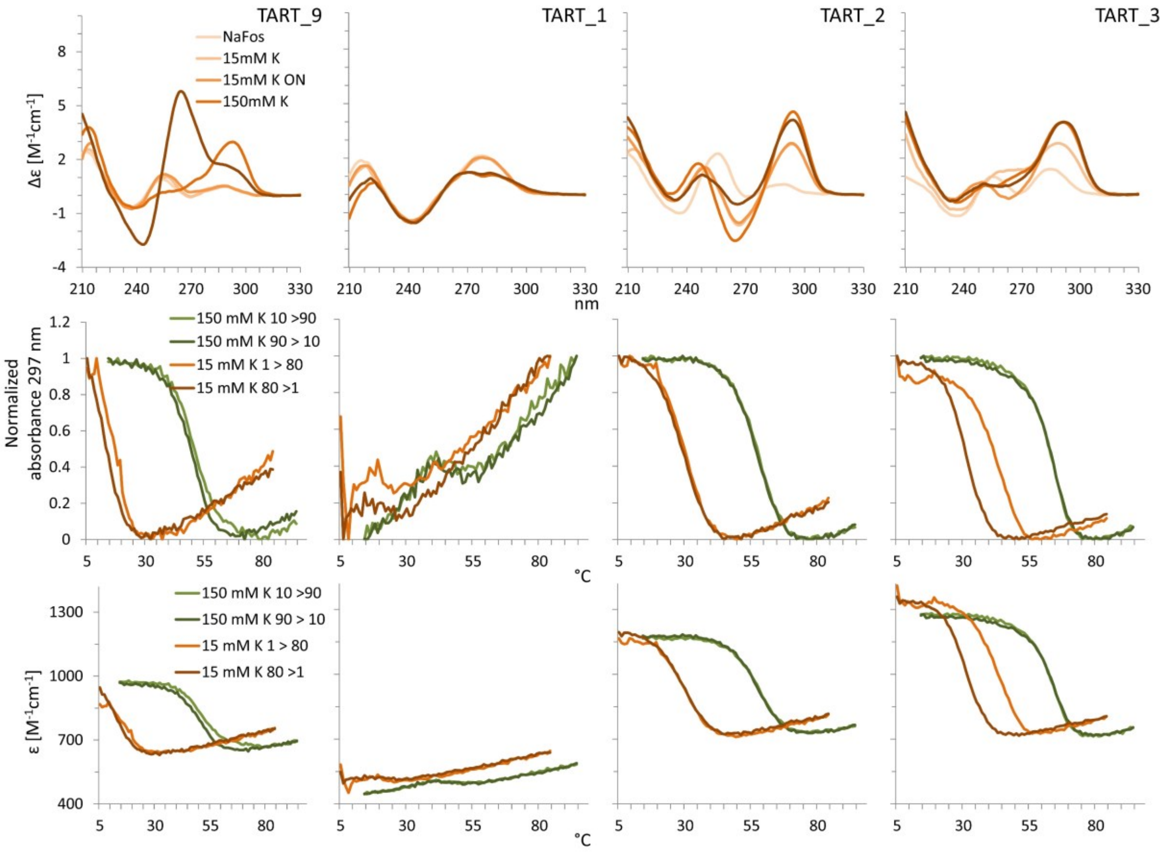
TART_9
TART_1
TART_2
TART_3
NaFos
15mM K
15mM K ON
150mM K
Δε [M-1cm-1]
nm
150 mM K 10 >90
150 mM K 90 > 10
15 mM K 1 > 80
15 mM K 80 >1
Normalized absorbance 297 nm
°C
ε [M-1cm-1]
°C

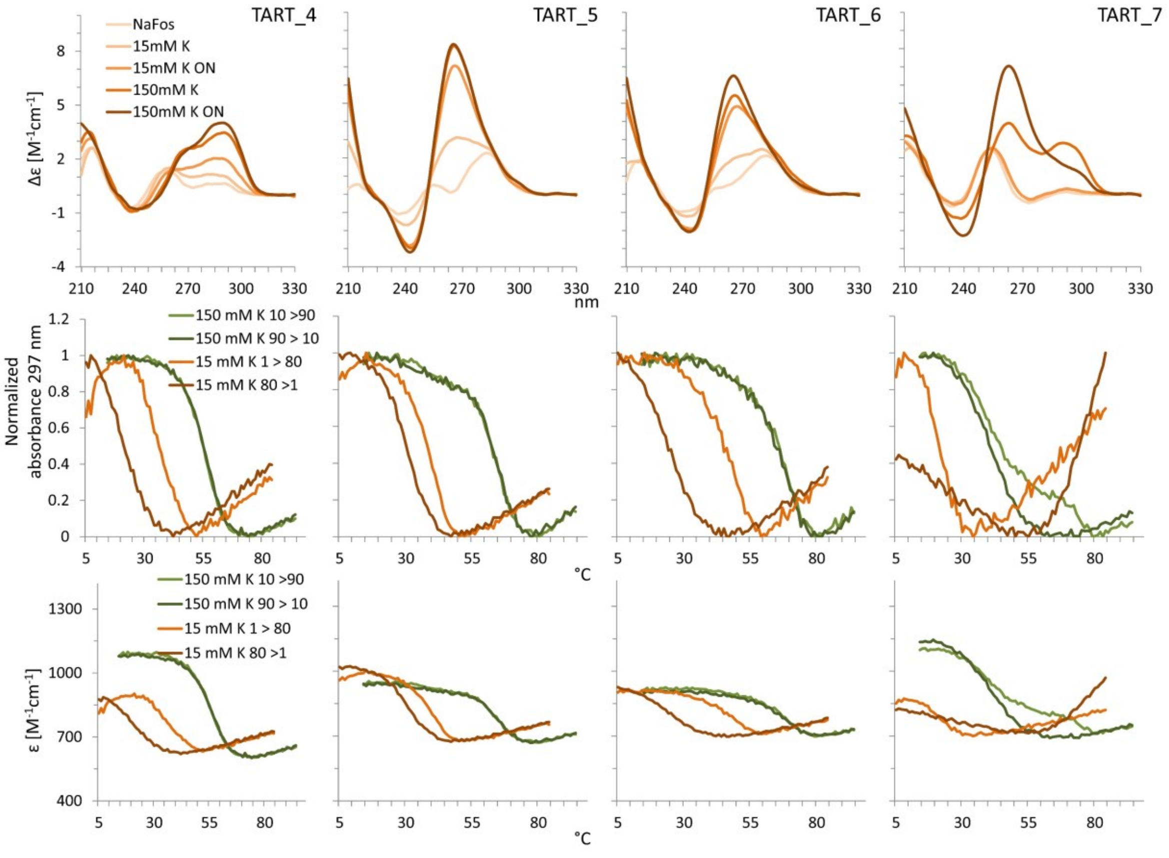
TART_4
TART_5
TART_6
TART_7
NaFos
15mM K
15mM K ON
150mM K
150mM K ON
Δε [M-1cm-1]
nm
150 mM K 10 >90
150 mM K 90 > 10
15 mM K 1 > 80
15 mM K 80 >1
Normalized absorbance 297 nm
°C
ε [M-1cm-1]
°C

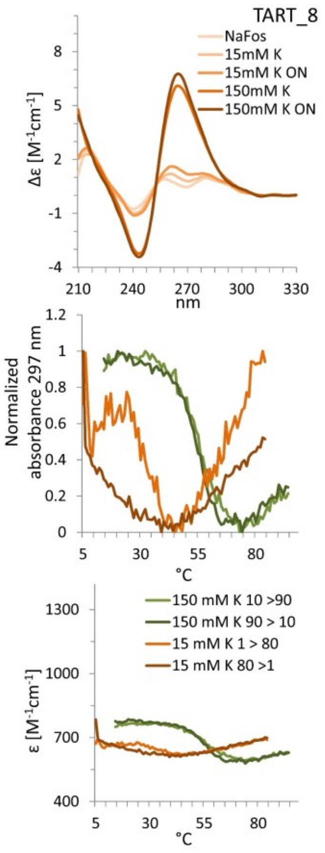
TART_8
NaFos
15mM K
15mM K ON
150mM K
150mM K ON
Δε [M-1cm-1]
nm
Normalized absorbance 297 nm
°C
150 mM K 10 >90
150 mM K 90 > 10
15 mM K 1 > 80
15 mM K 80 >1
ε [M-1cm-1]
°C

Figure S2. Proportions of repetitive DNA and PQS in *D. melanogaster* genome. The proportion of **A**) repetitive DNA and **B**) PQS in repetitive and non-repetitive DNA.

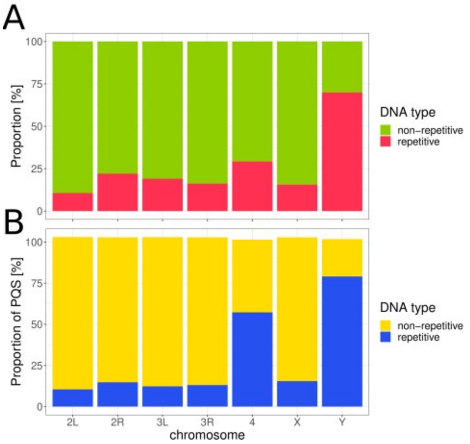

Figure S3. PQS proportion of Jockey clade elements in the genome of D. melanogaster. The PQS proportion is expressed as % of bps in all PQS containing fragments in the genome of a particular Jockey element.

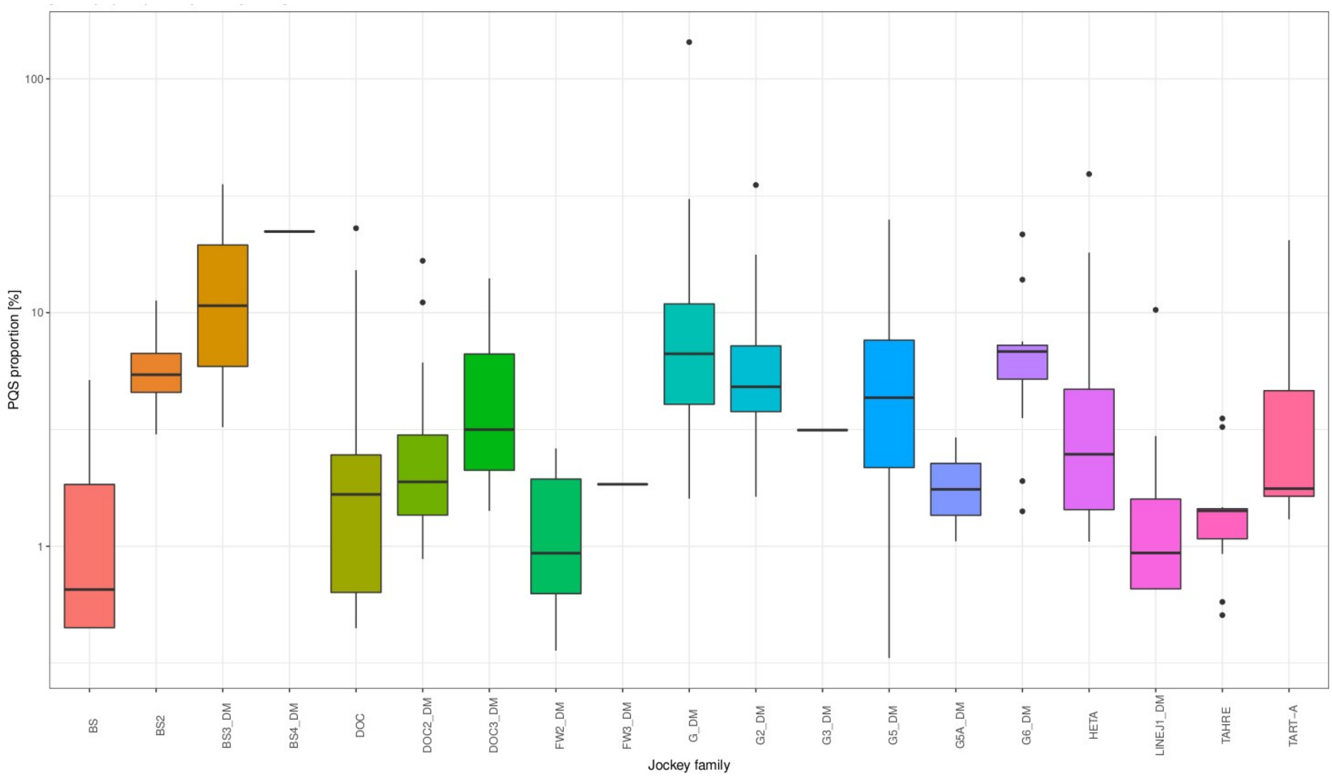

Figure S4. PQS abundance of telomeric retrotransposons in 14 Drosophila species. A) Phylogenetic relationships of telomeric retrotransposons based on RT and GAG domains. The colored division of the elements into 5 groups is based on a combination of both RT and GAG relationships. B) PQS abundance in individual elements of respective groups. NT_Jockeys stands for non telomeric Jockey elements of D. melanogaster.

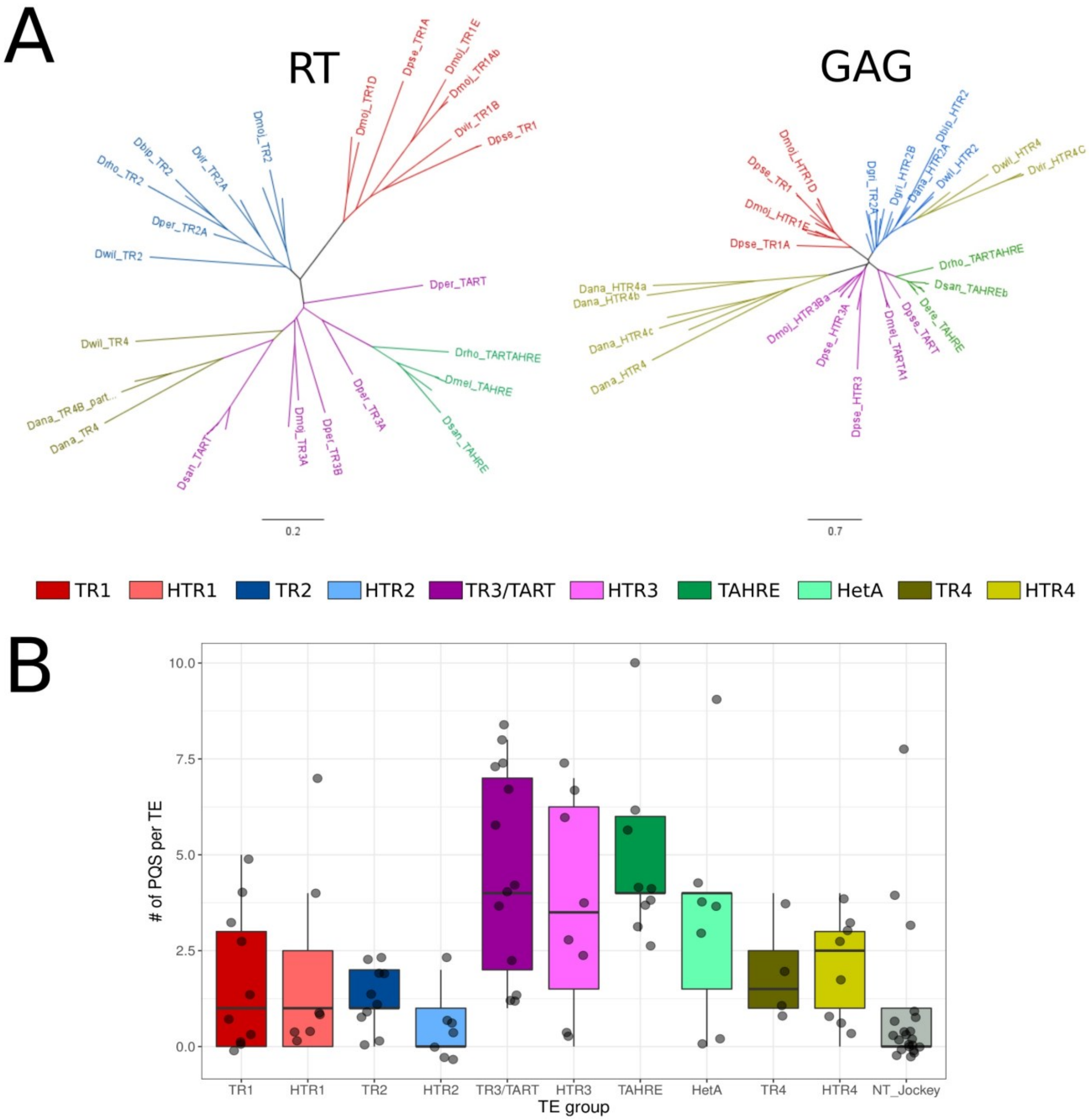

Figure S5. PQS density in telomeric retrotransposon arrays of the Drosophila genus. The order and taxonomic division of given species was adapted from the Drosophila genus consensus phylogenetic tree in Seetharam & Stuart (2013)

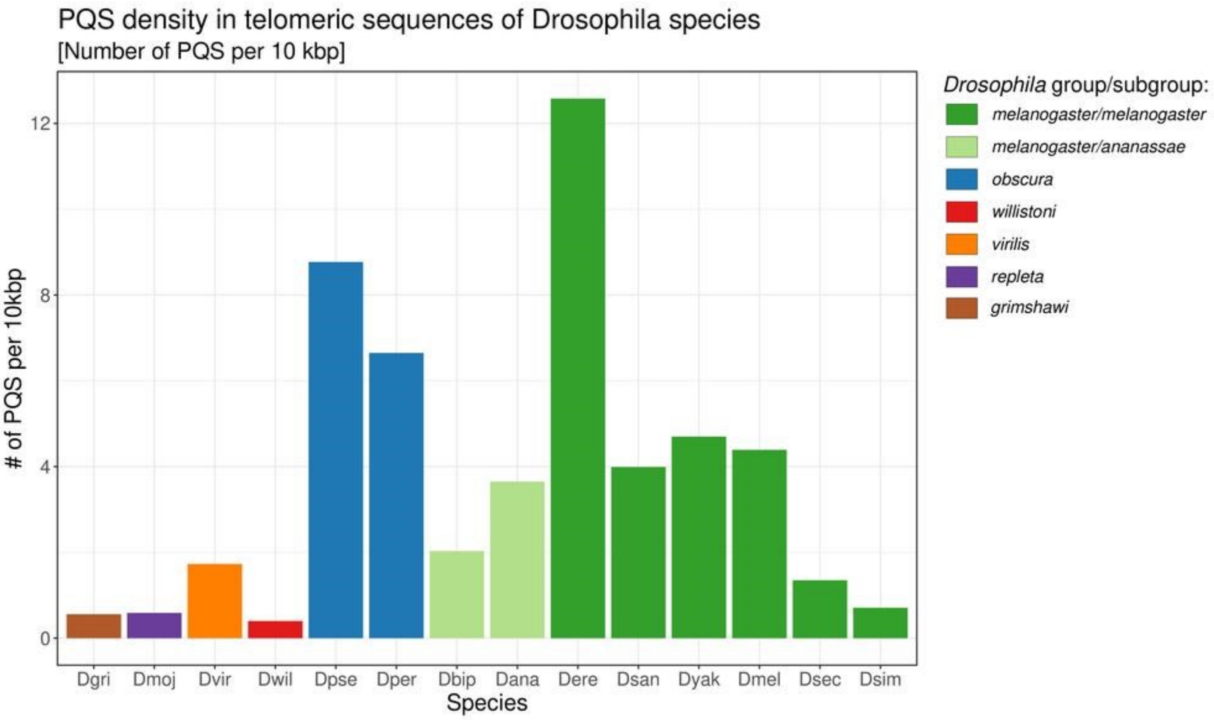

Figure S6: Sequence logos of conserved motives in GAG proteins from Drosophila species telomeric retrotransposons. A) Part of the alignment of all GAG proteins from telomeric elements, similarities are highlighted. The conserved region is indicated (dark-yellow arrow) and as well as 4 domains/motifs within the conserved region (pale-yellow arrows) for which sequence logos are shown below. Note that logos **B-D** are from alignment excluding TR4 elements. All PQS are indicated on consensus by purple boxes. **B)** PX(I/L)X7IXP(L/I)2X2(L/I)X10T(K/R)X25L motive **C)** major homology region - MHR (Rashkova et al, 2003) **D)** Pre-C2HC_pfam07530 and **E)** three zinc knuckle (C2HC) motives.

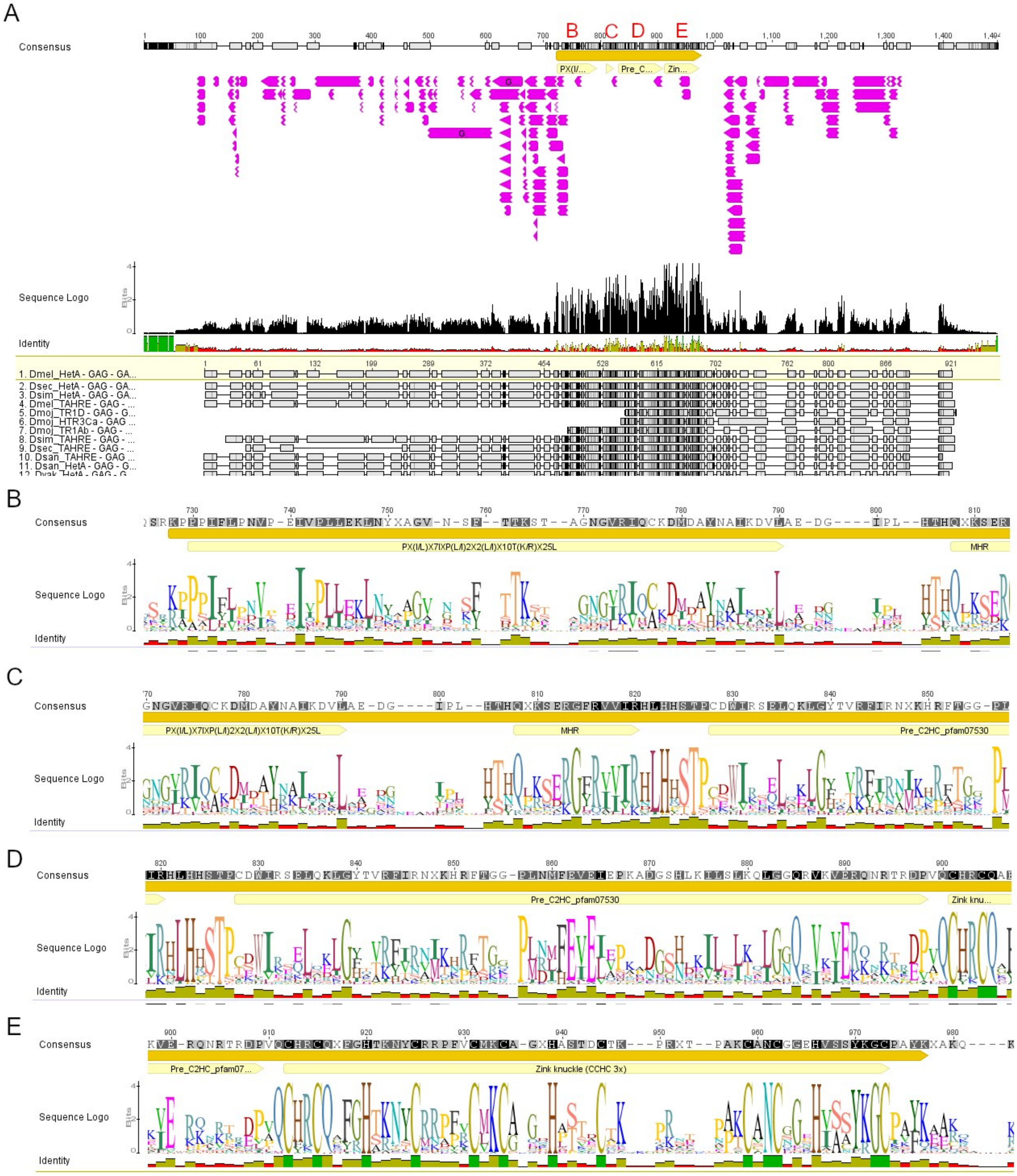

Figure S7. LINE elements show higher DNA conservation only if AA identity is used. Charts show comparison of average DNA and AA identity and similarity for gag and pol genes in several LINE groups as well as the DNA-AA identity and similarity difference. J1_D.sp and G5_D.sp are homologs of Dmel J1 and G5 Jockey elements that were found through BLAST search in Drosophila subgroup species. Jockey_Dmel represents a selection of telomeric as well as non-telomeric Jockey elements from the D. melanogaster genome. L1, R1 and CRE represent comparisons of various elements from respective groups.

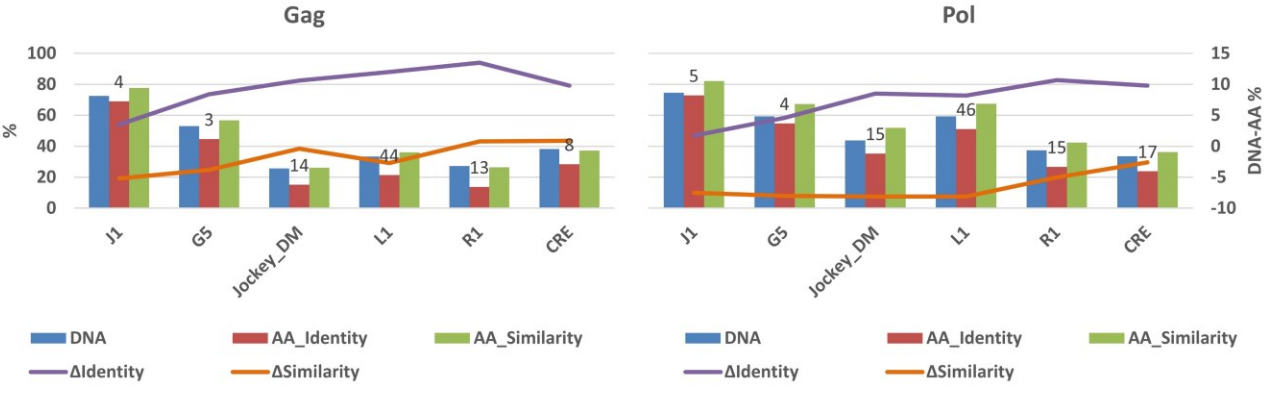

Figure S8. HTT elements show higher DNA conservation in gag 5´ region compared to J1 Charts show differences in DNA and AA identity and similarity in 3 regions of the gag gene in non-telomeric J1 elements as well as telomeric HTT elments form *D. simulans, D. sechellia, D. melanogaster* and *D. yakuba*. Genes for histone H3 and alcohol dehydrogenase (Adh) are included for compariison.

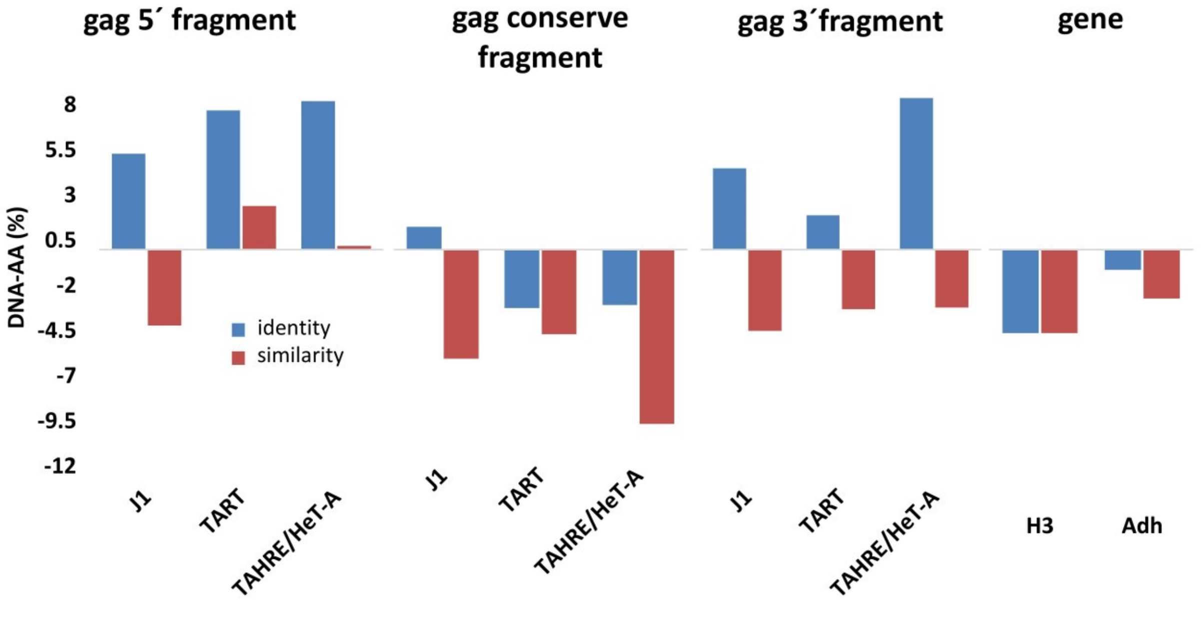

Figure S9. 5´ region of the gag gene show the highest dn/ds ratio Charts show ration of nonsynonymous and synonymous substitution rates in three gag regions as well as pol gene in telomeric retrotransposons in 14 *Drosophila* species.

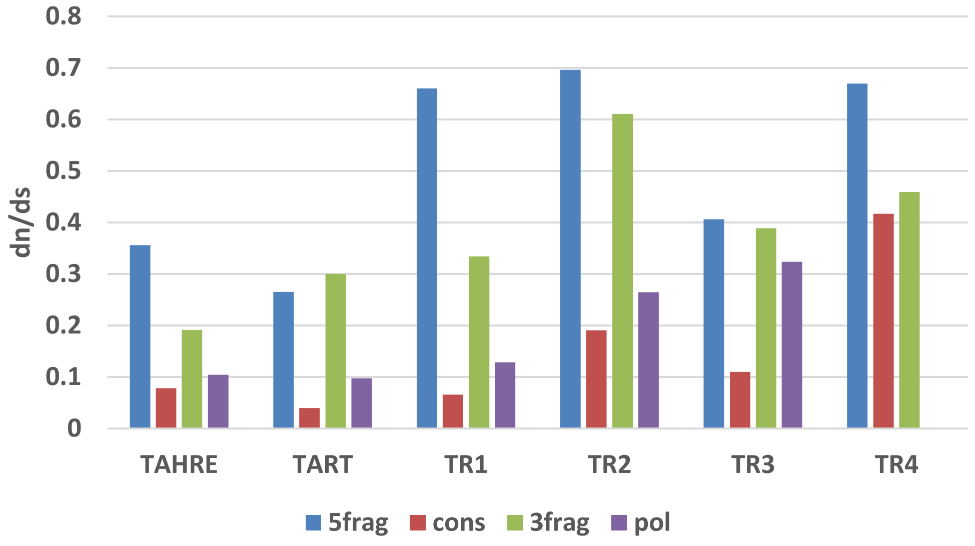

Figure S10. Delta GC content in GAG codons. Differences of GC content in codons given in respective GAG coding sequences and GC content in synonymous codons present in genetic code [%]. PQS loci are indicated by gray.

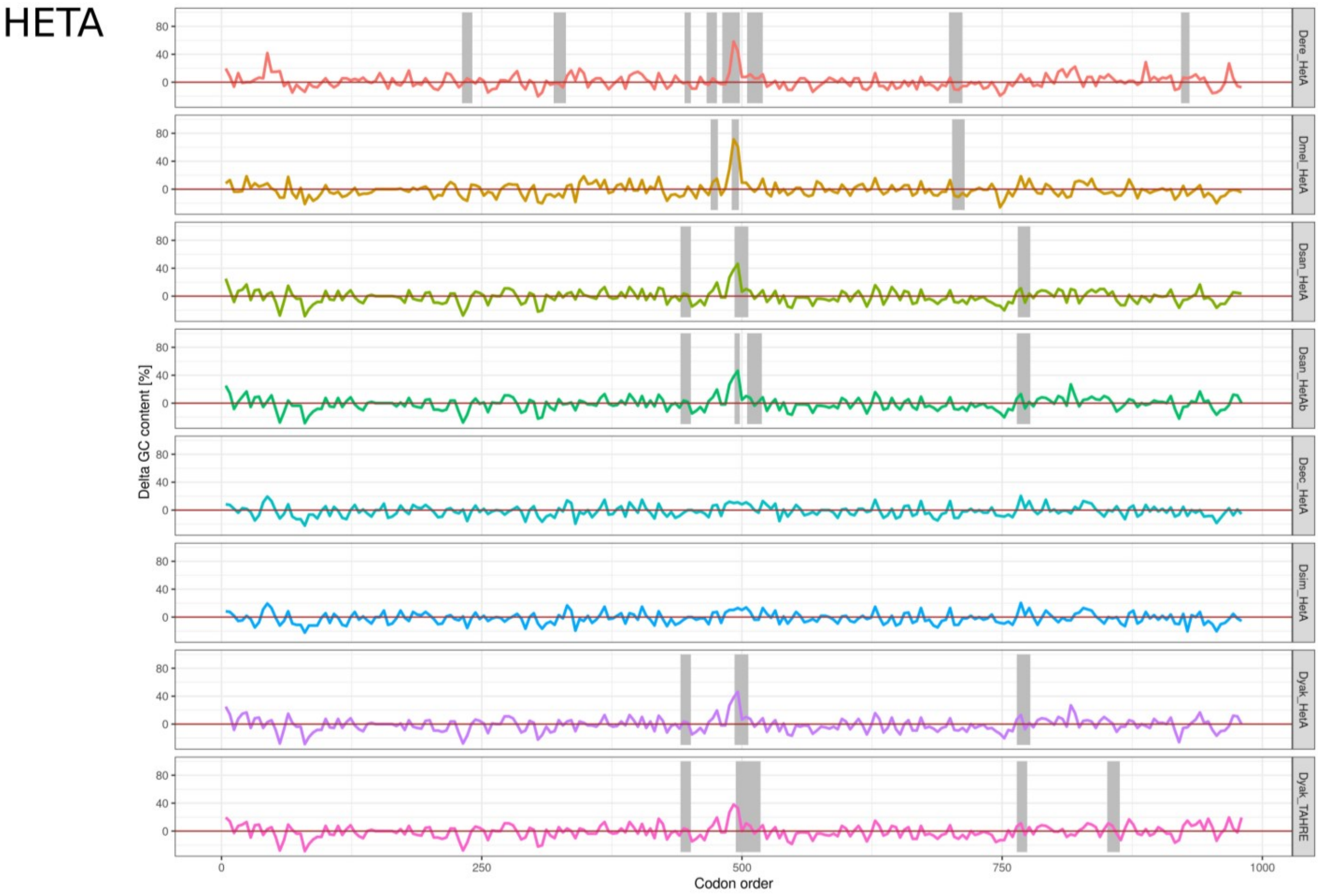

TAHRE

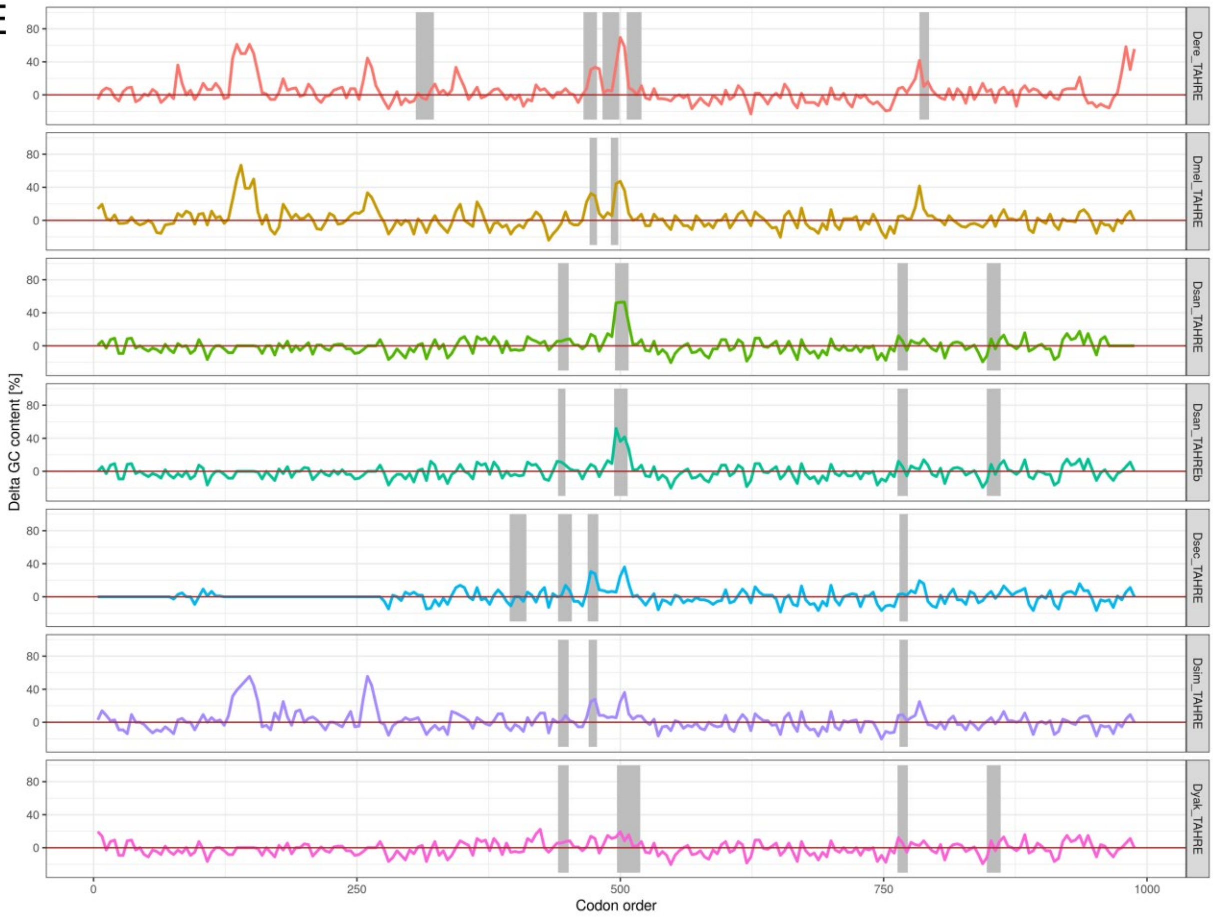
Dere_TAHRE
Dmel_TAHRE
Dsan_TAHRE
Dsan_TAHREb
Dsec_TAHRE
Dsim_TAHRE
Dyak_TAHRE
Delta GC content [%]
80
40
0
0
250
500
750
1000
Codon order

TART

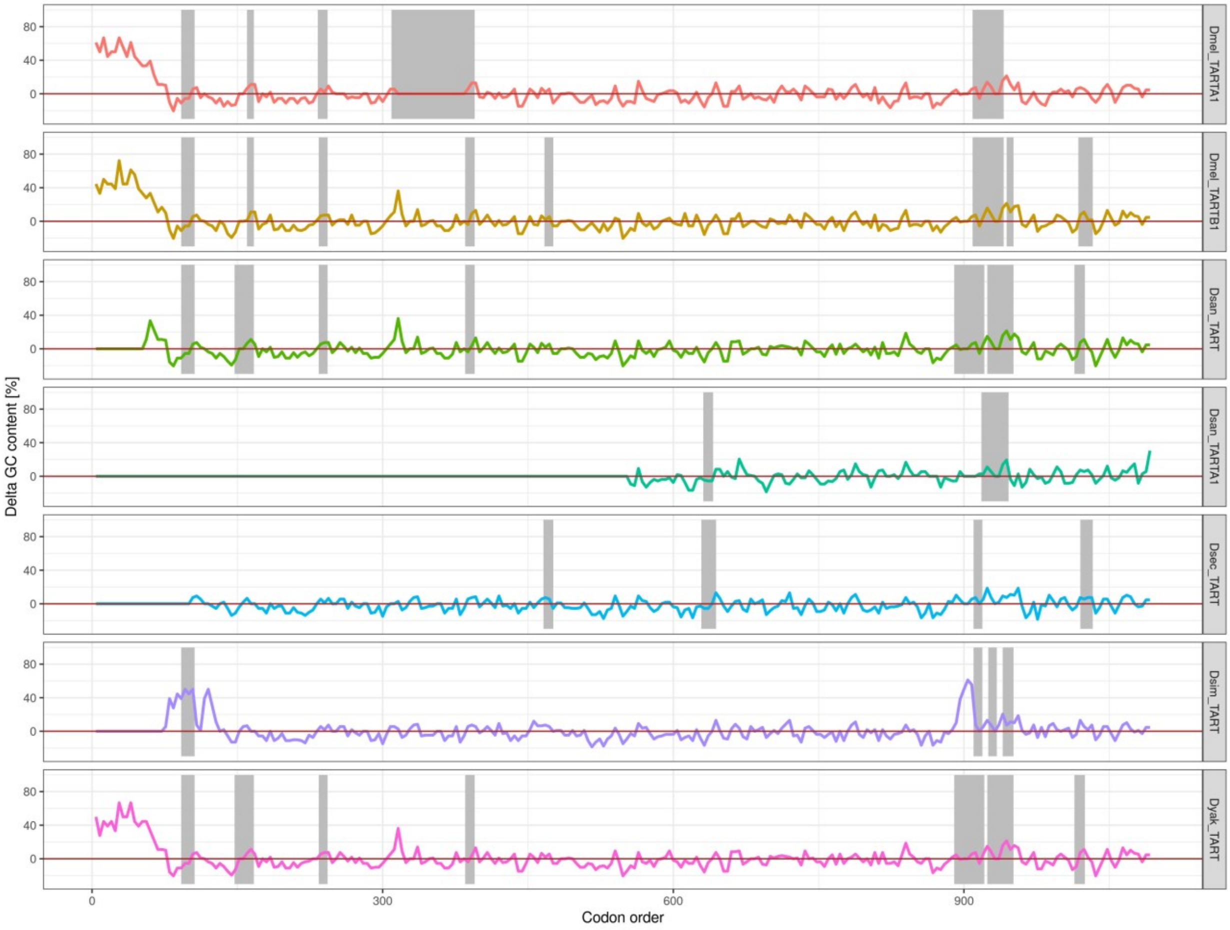
Dmel_TARTA1
Dmel_TARTB1
Dsan_TART
Dsan_TARTA1
Dsec_TART
Dsim_TART
Dyak_TART
Delta GC content [%]
80
40
0
0
300
600
900
Codon order

Figure S11. Comparison of PQS and non-PQS regions within GAG coding sequences of Drosophila telomeric retrotransposons. Preferential usage of (A) cytosine in third codon position and (B) amino acid (AA) coding . AA are presented in descendant order of their GC content in synonymous codons present in genetic code (e.g. Li et al., 2015)

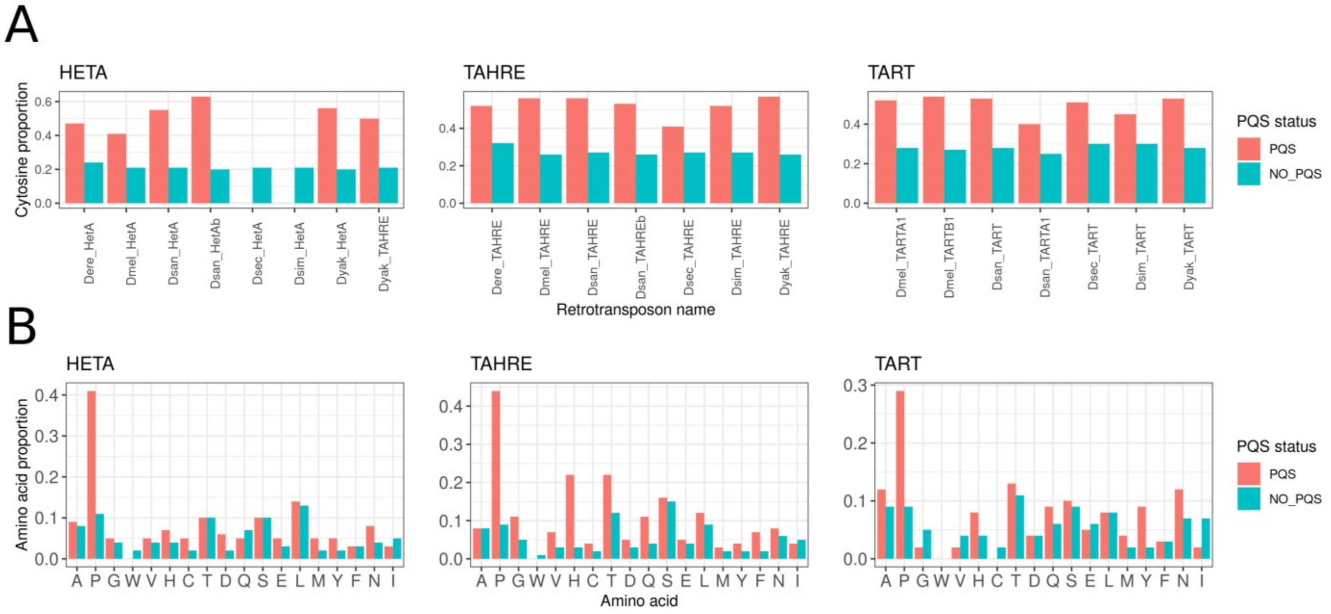

Figure S12. RT-based phylogram of representative LINE elements showing that GilM/GilT elements belong to the CRE-like elements. All telomere-associated elements are bolt and the respective LINE clades are highlighted.

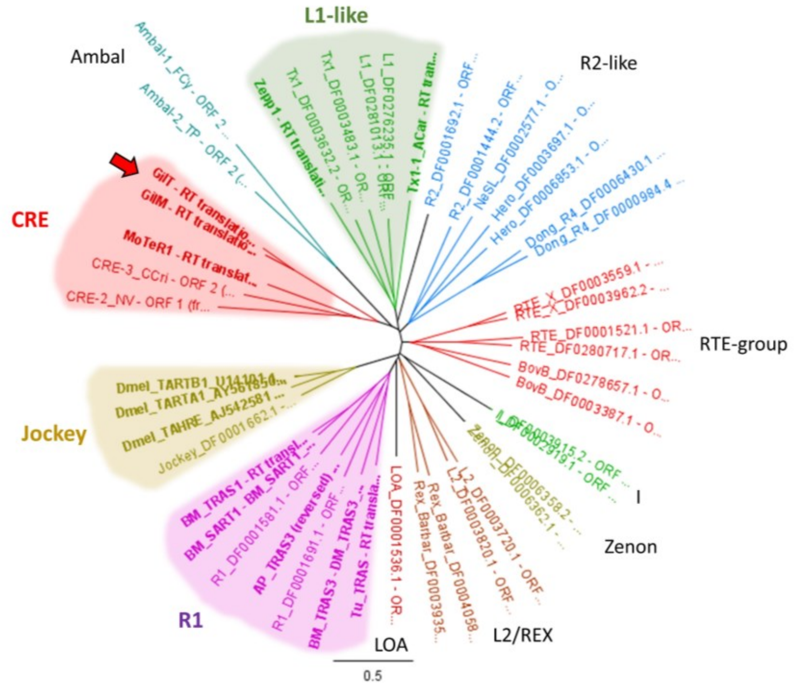

References:

Abad JP, Villasante A. The 3 non-coding region of the Drosophila melanogaster HeT-A telomeric retrotransposon contains sequences with propensity to form G-quadruplex DNA. FEBS Lett.1999;453:59–62.

Li J, Zhou J, Wu Y, Yang S, Tian D. GC-Content of Synonymous Codons Profoundly Influences Amino Acid Usage. G3 (Bethesda).2015;5:2027-36.

McGurk MP, Dion-Côté AM, Barbash DA. Rapid evolution at the Drosophila telomere: transposable element dynamics at an intrinsically unstable locus. Genetics 2021, 217(2), iyaa027.

Rashkova S, Athanasiadis A, Pardue ML. Intracellular targeting of Gag proteins of the Drosophila telomeric retrotransposons. J. Virol.2003;77:6376–6384.

Seetharam AS, Stuart GW. Whole genome phylogeny for 21 Drosophila species using predicted 2b-RAD fragments. PeerJ.2013;1:e226.
